# Floodplain nitrifiers harbor the genetic potential for utilizing a wide range of organic nitrogen compounds

**DOI:** 10.1128/msystems.00829-25

**Published:** 2025-10-13

**Authors:** Anna N. Rasmussen, Katie Langenfeld, Bradley B. Tolar, Zach Perzan, Kate Maher, Emily L. Cardarelli, John R. Bargar, Kristin Boye, Christopher A. Francis

**Affiliations:** 1 Stanford Synchrotron Radiation Lightsource, SLAC National Accelerator Laboratory 17220, Menlo Park, California, USA; 2 Department of Earth System Science, Stanford University 539510, Stanford, California, USA; 3 Environmental Molecular Sciences Laboratory, Pacific Northwest National Laboratory 6865, Richland, Washington, USA; 4 Oceans Department, Stanford University 6429, Stanford, California, USA; University of California, Davis, Davis, California, USA

**Keywords:** nitrification, metagenomics, floodplain, sediments, nitrogen metabolism, organic nitrogen

## Abstract

Organic compounds such as urea and cyanate can serve as nitrogen (N) sources for nitrifying microorganisms, including ammonia-oxidizing archaea (AOA) and bacteria (AOB), complete ammonia-oxidizing (comammox) bacteria, and nitrite-oxidizing bacteria (NOB). Here we investigated metagenome-assembled genomes (MAGs) for all four nitrifier guilds generated from hydrologically variable floodplain sediments of the Wind River Basin (WRB; Riverton, WY, USA) for their genetic potential to utilize organic N compounds. A vast majority of WRB nitrifier MAGs harbored urease (*ure*) and at least one urea transporter (*utp*, *urt*, *dur3*). AOA were the most abundant and phylogenetically diverse nitrifiers in WRB floodplain sediments. Several AOA MAGs encoded cyanase (*cynS*), nitrilase (*nit1*), omega-amidase (*nit2*), nitrile hydratase (*nthA*), and genes related to purine degradation, including biuret hydrolase (*biuH*), oxamic transcarbamylase (*allFGH*), and catabolic carbamate kinase (*allK*). AOA often encoded an uncharacterized amidohydrolase collocated with *biuH*, rather than allophanate hydrolase (*atzF*). A small number of AOA encoded *atzF*, functioning in an unknown pathway. AOB and comammox were of relatively low abundance and taxonomic diversity and were present only at certain depths in WRB; however, they encoded triuret/biuret degradation genes (*trtA*, *biuH*, and *atzH*), and in comammox, these genes were also collocated with *allFGHK*. The genetic potential of ammonia oxidizers in the WRB floodplain suggests that organic N may support nitrification in this system. The proposed pathways for utilizing purine degradation products other than urea potentially expand the known metabolic capabilities of AOA, AOB, and comammox bacteria and reveal the possibility for cryptic N cycling between microbial community members.

**IMPORTANCE:**

Floodplains are critical ecosystems where terrestrial and riverine systems meet. Floodplain sediments experience many, sometimes dramatic, changes in moisture and oxygen concentrations because of changes in water table height, flooding, and drought, leading to active microbial cycling of contaminants and nutrients. Nitrogen is one such nutrient that is not only essential for the building blocks of life but can also be used as an energy source by some microorganisms. Microorganisms that oxidize ammonia and nitrite are a crucial part of the nitrogen cycle and can lead to eventual nitrogen loss from a system. Investigating the genes present in microorganisms responsible for nitrification in a dynamic floodplain suggests that organic nitrogen—from decaying plants or potentially other sources, such as fertilizers, grazing livestock feces, or contaminants (e.g., pesticides, pharmaceuticals)—is an important nitrogen source to these microorganisms. This study identifies genes not previously described in nitrifying microorganisms, expanding their potential metabolic substrates.

## INTRODUCTION

Floodplains are dynamic ecosystems where riverine and terrestrial systems intersect. Floodplains experience hydrological shifts, such as changes in water table height, flooding, and drought. The resulting fluctuations in sediment moisture and saturation typically drive key geochemical transformations and subsurface exchanges of water, nutrients, and other compounds across different sediment layers (1–7). Floodplains can contain high concentrations of organic nitrogen (N), and the cycling of this N is important as N is an essential element for life, can be a limiting nutrient, or become a pollutant at high concentrations (8). Nitrification, the oxidation of ammonia to nitrate via nitrite, is a critical step in the N cycle linking fixed N to N-loss processes. There are several guilds of microorganisms responsible for nitrification, including ammonia-oxidizing archaea (AOA), ammonia-oxidizing bacteria (AOB), complete ammonia-oxidizing bacteria (comammox), and nitrite-oxidizing bacteria (NOB).

In the western USA, the prevalence of nitrifier populations, particularly ammonia oxidizers, has recently been investigated in a wide range of floodplain systems. A metagenomic and metatranscriptomic study of floodplain sediments of the East River (ER; near Crested Butte, CO, USA) found genes encoding nitrification enzymes (ammonia monooxygenase and nitrite oxidoreductase) to be some of the most highly transcribed genes despite recovering a low diversity of nitrifier metagenome-assembled genomes (MAGs) (9). In the neighboring Slate River (SR; near Crested Butte, CO, USA), comammox, oligotrophic-adapted AOA, and a putative NOB sister clade to the *Nitrospiraceae* were the numerically dominant nitrifiers present in MAGs from floodplain sediments (10). A study of ammonia monooxygenase subunit A (*amoA*) genes across five floodplain sediment profiles spanning a 900 km north–south transect of the intermountain western USA (WY, CO, NM, US) found AOA to be incredibly diverse and numerically dominant over AOB (11). Based on 16S rRNA gene amplicon libraries from Wind River Basin (WRB; near Riverton, WY, USA) floodplain sediments, microbial communities include diverse AOA and AOB populations and are generally structured by depth, remaining persistent over time despite seasonal changes in water table height (12). The AOA communities in WRB floodplain sediments are structured by both sediment moisture (11, 13) and depth-associated changes in lithology (12). Beyond the USA, several studies in the Amazon River floodplain have proposed a link between nitrification and N-dependent methanotrophy (14–16), creating further potential links between N and carbon cycling.

Nitrifying microorganisms use N as both an energy source and for building biomass, and some can utilize organic N compounds to meet their N needs. Urea and cyanate can both directly and indirectly provide N for ammonia oxidizers (17–20) and nitrite oxidizers (21). Degradation products from organic N compounds can be exchanged between different guilds of nitrifiers through reciprocal feeding (i.e., between ammonia and nitrite oxidizers) and cross-feeding with other microbial community members. For example, NOB catabolizes cyanate or urea to meet cellular N-demand (17) and may also supply ammonia to ammonia oxidizers, which in turn produce nitrite that can be oxidized by NOB (18, 22). A recent study also proposes cryptic N-cycling in oceans between abundant microbial community members, where *Prochlorococcus* and AOA exude purines and pyrimidines that are degraded by SAR11 to urea, which can then serve as a N-source for AOA, *Prochlorococcus*, and other microbial community members (23). Remineralization of polyamines by heterotrophic community members also likely allows putrescine-derived N to support nitrification in marine environments (24).

The metabolic potential to utilize organic N can benefit nitrifiers in several ways, by allowing them to thrive in environments with low ammonia concentrations or by creating separate niches for different nitrifier guilds. For example, urea-fed systems can allow the co-existence of comammox and NOB *Nitrospira*, or AOA and AOB, compared to systems with high ammonia amendments (25, 26). Urea and cyanate degradation pathways are encoded by many nitrifiers: for example, a comparative genomics analysis of 289 high-quality genomes found 50% of AOA, 60% of AOB, and 80% of comammox encoded genes for urea transport, urease, and accessory proteins (27). In another study of 70 representative nitrifier genomes, ~50% encoded genes related to urea uptake and degradation, and ~12% encoded cyanate lyase (*cynS*), while known genes involved in the degradation of polyamines, taurine, glycine betaine, and methylamines were completely absent (28). However, the possible organic N sources directly used by nitrifiers continue to expand to include sewage-, farming-, and industrial-related contaminants, as well as other organic matter breakdown products. For example, a recent study shows that some comammox, and perhaps AOB, can use guanidine—a nitrogen-containing compound formed by degradation of compounds such as guanylurea, metformin, and cyanoguanidine—as a reductant, an energy source, and an N source (29). A putative nitrilase (NIT1) is mostly conserved in AOA (30), while a nitrile hydrolase (NTH) (13) and a putative nitrilase/omega amidase (NIT2) (30) are encoded by some AOA, supporting their potential degradation of nitriles—a broad class of naturally occurring and man-made organic compounds containing a cyano functional group—and/or dicarboxylic acid monamides to produce ammonia. Another recent study found the genetic potential in comammox bacteria for degrading polyurea molecules, triuret (carbonyldiurea), and biuret (carbamylurea) (10), which are common impurities in urea fertilizers and breakdown products from oxidative purine and uric acid degradation (31–35). Laboratory incubations of forest soils have found that triuret- and biuret-derived N can support nitrification, albeit at much slower rates than for urea-fertilized soils (36–38).

**Fig 1:**
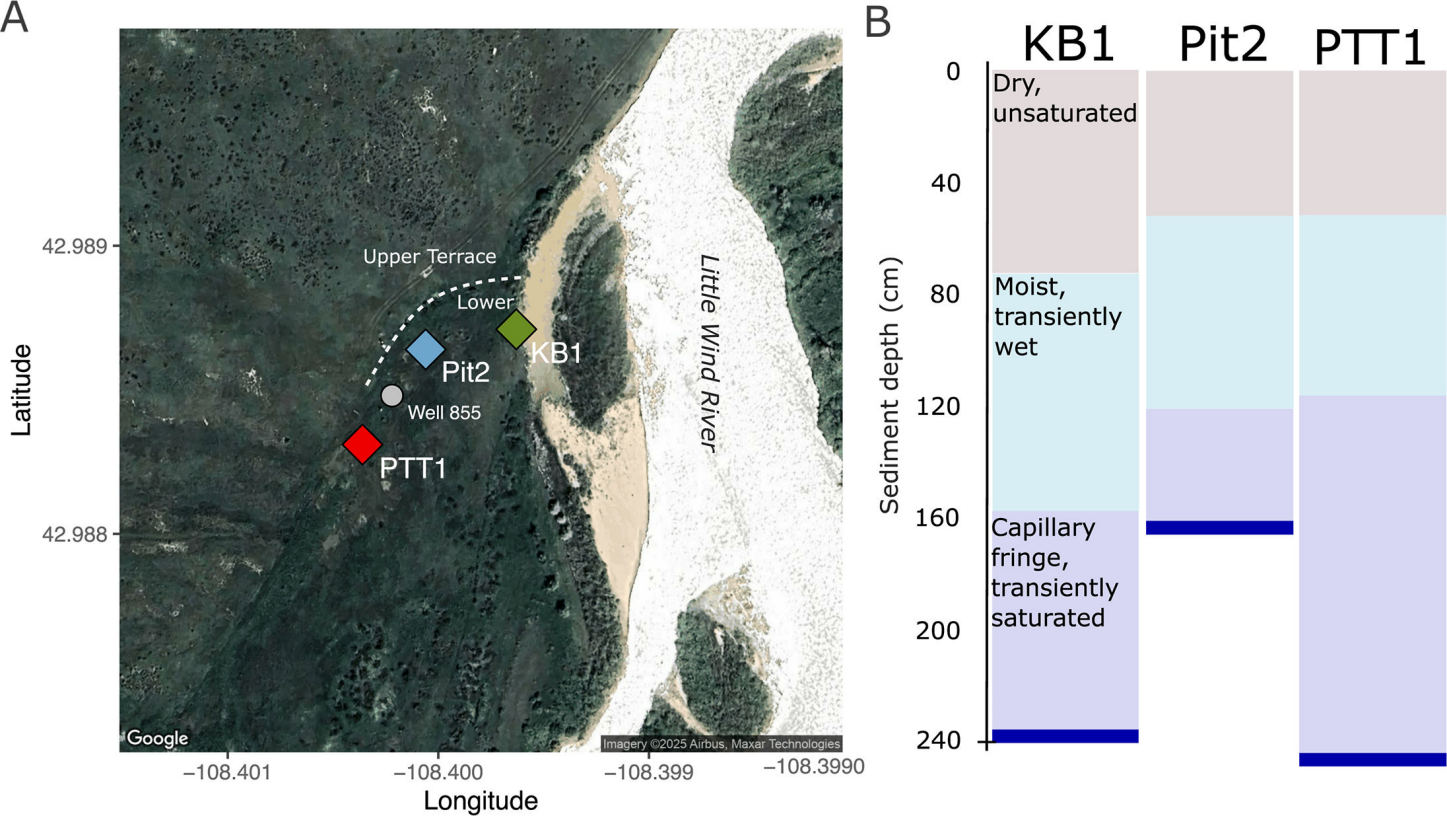
(**A**) Map of sampling sites in the Wind River Basin (WRB) near the Little Wind River and Wind River, Wyoming, USA (Map data ©2025 Google ©2025 Airbus, Maxar technologies). (**B**) Schematic of general sediment conditions at each site.

Systematic studies of environments rich in organic nitrogen but with potentially variable inorganic N sources, such as natural floodplains (39–41), can help identify which organic N compounds may be used directly by nitrifying microorganisms. In this study, we investigate nitrifier diversity and genetic potential to utilize alternative sources of ammonia in hydrologically variable floodplain sediments. We analyzed MAGs generated from the WRB at three sites (KB1, Pit2, and PTT1; Fig. 1) with generally similar sediment geochemical composition but varying depths to gravel bed/capillary fringe, sampled in different years (2015, 2017, and 2019, respectively).

## RESULTS

**Fig 2:**
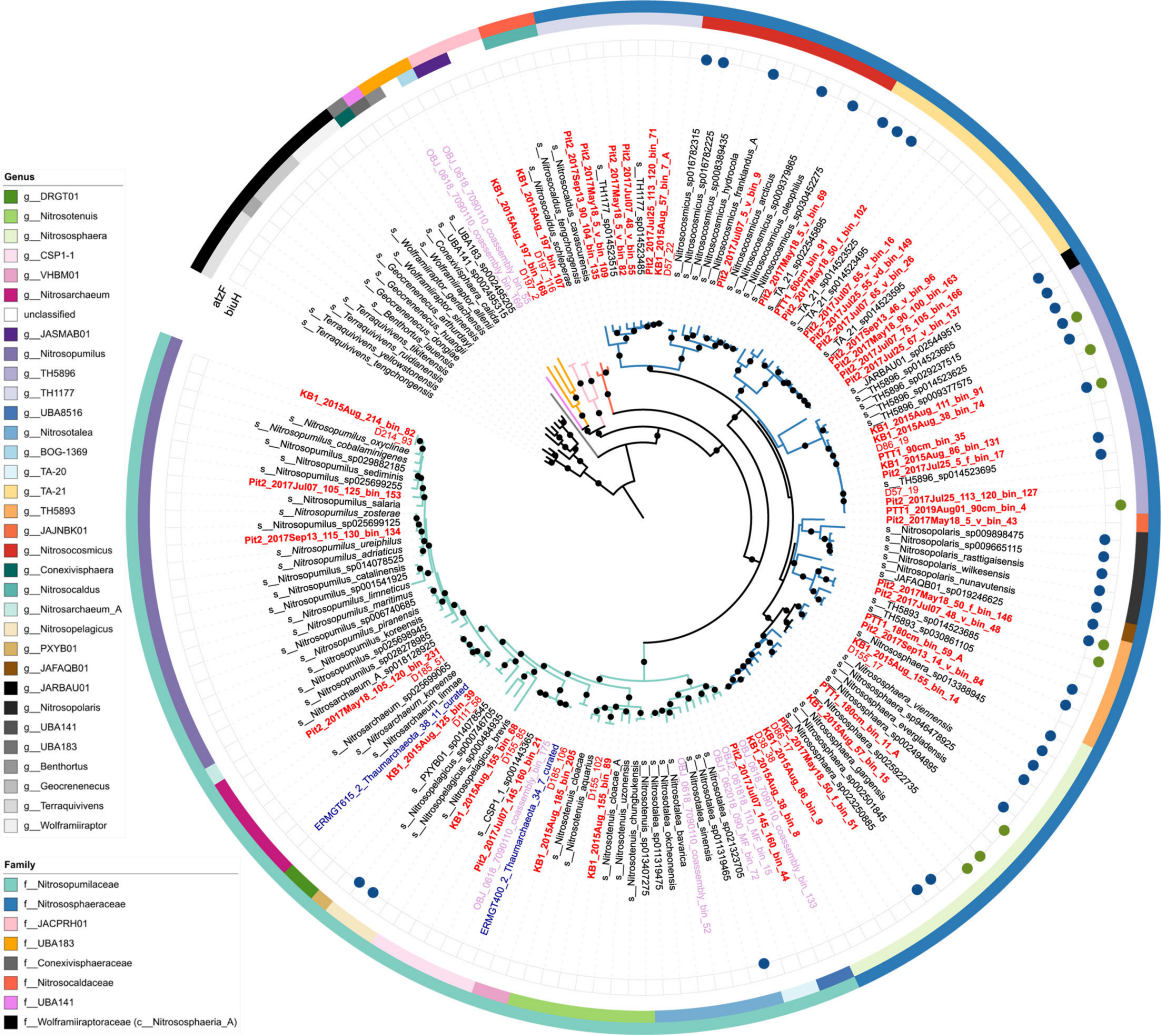
Concatenated ribosomal tree made using IQ-TREE2 with model LG + F + R7 for select *Nitrososphaerales* MAGs, including all non-redundant plus *atzF*- or *biuH*-encoding *Nitrososphaeria* MAGs generated from WRB (red), Slate River (SR; light purple), and East River (ER; blue), as well as NCBI type material (italics) and *atzF*- or *biuH*-encoding GTDB species representatives. MAGs generated in this study are in bold. External circles indicate the presence of the *biuH* and *atzF* genes. Outer ring color indicates GTDB-assigned family, and the inner ring indicates genus. Black dots indicate nodes with ≥ 90% bootstrap support. Tree rooted with *Nitrososphaeria_A*.

**Fig 3:**
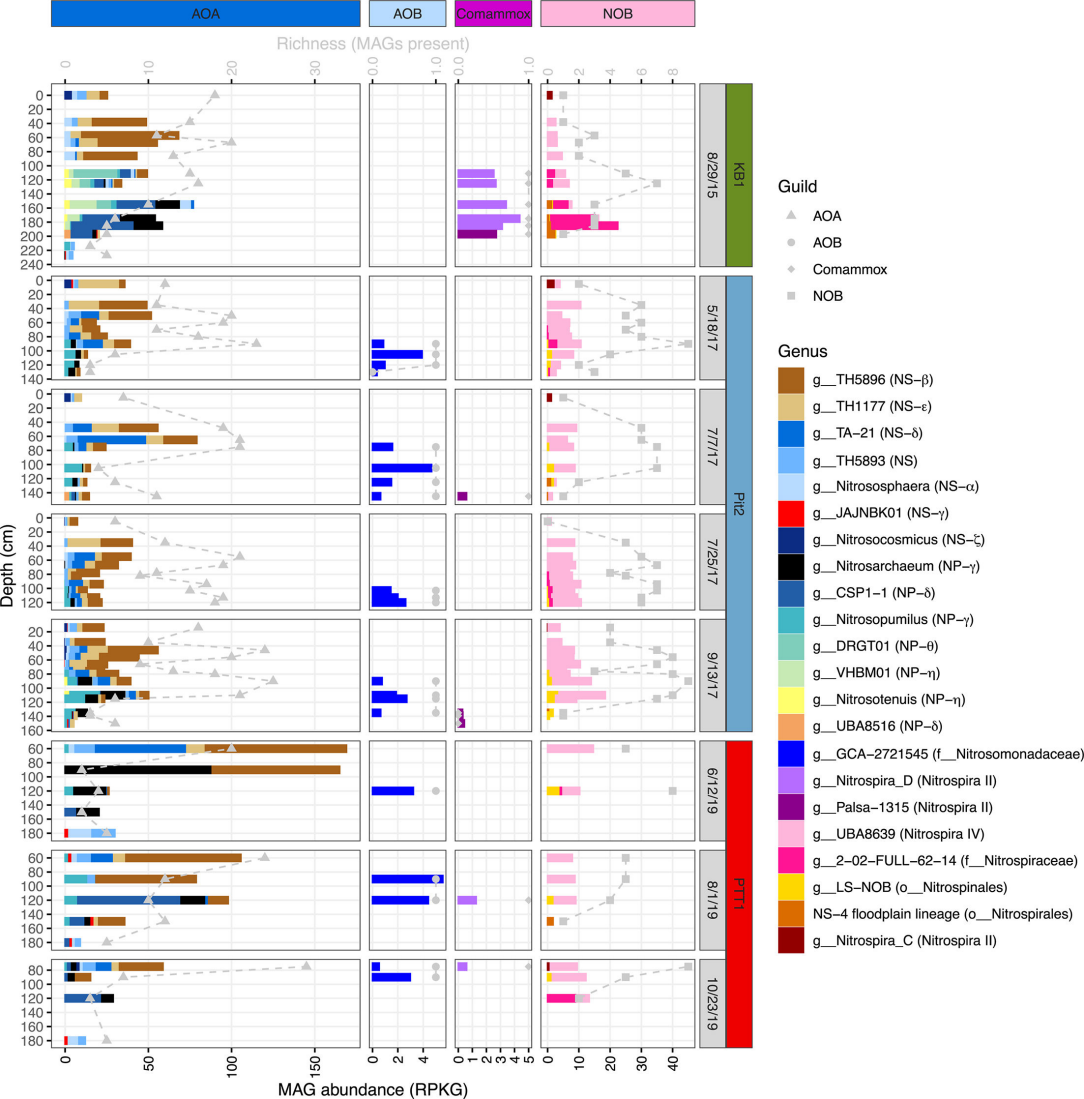
Relative abundance and richness of nitrifier genera (x-axis) at each depth sampled (y-axis). Relative abundance is displayed as bar plots in reads per kilobase of genome per gigabase of metagenome (RPKG), and richness is displayed as gray dashed lines and points in observed MAGs present. Both the x-axis and y-axis vary between panels. The relevant order, family, *Nitrospira* lineage, or AOA *amoA*-defined subgroup (42; NP = *Nitrosopumilaceae*, NS = *Nitrososphaeraceae*) is indicated in the parenthesis next to the genus names.

A total of 9,989 MAGs (bins > 50% complete and <10% contamination) were generated from 68 floodplain metagenomes from three nearby sites in the WRB. After dereplication at 98% average nucleotide identity (ANI), a total of 3,874 MAGs formed a non-redundant data set of microbial “lineages” present in the site. The non-redundant MAG data set recruited 13.5%–57.4% (mean 43.8%) of metagenome reads, with the lowest recruitment occurring in the shallowest samples (Fig. S1). MAGs originated from over 60 phyla, with the highest number coming from *Actinomycetota* (formerly *Actinobacteria*, *n* = 2,066) and *Pseudomonadota* (formerly *Proteobacteria*, *n* = 2,065). MAGs for several nitrifier guilds were recovered, including AOA (*n* = 189), AOB (*n* = 11), comammox bacteria (*n* = 6), and NOB (*n* = 68). Additionally, two non-AOA *Nitrososphaeria* MAGs similar to those reported previously (13) were recovered from the “JACPRH01” family (*Nitrososphaerales*) (Fig. 2). No anammox MAGs were recovered. All together, 63 nitrifier lineages spanning 22 genera were recovered. Nitrifiers were present in 67 of 68 samples at an average abundance of 44 reads per kilobase of genome per gigabase of metagenome (RPKG; range = 4.5 to 184 RPKG). Nitrifier richness (total number of observed MAGs present) at each site was similar (*n* = 51, 53, and 56 at PTT1, KB1, and Pit2, respectively); however, nitrifier richness per sample (median = 16) varied by depth with the deepest sediments having the lowest richness (Fig. 3).

Nitrifier distribution was structured by depth and site in both unconstrained and constrained ordinations (Fig. S2). In a Constrained Analysis of Principal Coordinates (CAP), depth and site explained 24.3% of community variation (ANOVA, *P* < 0.001) with depth having the larger marginal effect (14.2%, ANOVA, *P* < 0.001) followed by site (10.1%, ANOVA, *P* < 0.001) (Fig. S2B and C). Despite some site heterogeneity in nitrifier communities, similar distribution patterns at the family and guild level were observed at all three sites (Fig. S2). Generally, shallower depths (0 to ~100 cm) were numerically dominated by AOA from the *Nitrososphaeraceae* family (1,731 RPKG) and NOB from the “UBA8639” family (*Nitrospira* lineage IV; 330 RPKG), in contrast to deeper depths (~100 cm to 234 cm), which contained a broader diversity of nitrifiers at the family and guild levels, including AOA from the *Nitrosopumilaceae* (741 RPKG); established and putative NOB from the *Nitrospiraceae* (78 RPKG), *Nitrospinales* (31 RPKG), and “NS-4” (*Nitrospirales*; 8 RPKG); comammox from both *Nitrospira_D* and “Palsa-1315” (*Nitrospiraceae*; 22 RPKG); and AOB from the *Nitrosomonadaceae* (43 RPKG; Fig. S2).

### Nitrifier taxonomic diversity

AOA were the most abundant and diverse in terms of richness of the nitrifiers throughout the sediment column at all three sites sampled (Fig. 3). We recovered medium- to high-quality AOA MAGs representing 45 lineages from 14 genera after dereplication at 98% ANI (Table S1). AOA were depth differentiated, with organisms from the *Nitrososphaeraceae* numerically dominating in the upper (< ~100 cm) sediment depths transitioning to *Nitrosopumilaceae* numerically dominating in deeper (> ~100 cm) depths (Fig. S2). The most abundant and widely distributed of the 7 *Nitrososphaeraceae* genera included “TH5896” (911 RPKG), “TH1777” (301 RPKG), “TA-21” (279 RPKG), and “TH5893” (125 RPKG) (Fig. 3). Of the low-abundance *Nitrososphaeraceae*, *Nitrososphaera* (82 RPKG) were cosmopolitan across the sediment column, in contrast to *Nitrosocosmicus* (15 RPKG) and “JAJNBK01” (18 RPKG), which were generally present only in the shallowest or deepest sediments, respectively. *Nitrosopumilaceae* was most abundant and had the highest richness at KB1, with the genera “DRGT01” and “VHBM01” present only at KB1. Among the *Nitrosopumilaceae* genera, *Nitrosarchaeum* was the most abundant overall (285 RPKG) and was found at all three sites; however, at both KB1 and PTT1, “CSP1-1” (234 RPKG) was the most abundant at deeper depths, in contrast to Pit2, where *Nitrosopumilus* (82 RPKG) was the most abundant (Fig. 3).

NOB were also cosmopolitan in WRB floodplain sediments (Fig. 3). Established and putative NOB MAGs originated from five genera from the *Nitrospirales* and *Nitrospinales* orders, represented by 15 lineages after dereplication. The vast majority (75%) of NOB MAGs originated from the Genome Taxonomy Database (GTDB)-defined genus UBA8639 within the UBA8639 family (*Nitrospira* lineage IV). The UBA8639 genus was present at most depths throughout the floodplain sediment column at all three sites and consisted of 11 lineages after dereplication with different depth distributions, including one lineage that was absent from KB1 (Fig. S3). Other established NOB include LS-NOB (*Nitrospinales*) that was present in deep sediments and *Nitrospira_C* (*Nitrospira* lineage II) that was present in shallow sediments. Uncultured, putative NOB were present in deeper depths and originated from “2-02-FULL-62-14” (*Nitrospiraceae*) and the same NS-4 (*Nitrospirales*) lineage found in SR floodplain sediments (Fig. S4) (10). In contrast to AOA and NOB, bacterial ammonia oxidizers were present at low abundance, occurred only at deeper depths, and consisted of a single lineage of comammox clade A (*Nitrospira*_D; *Nitrospira* lineage II), comammox clade B (Palsa-1315; *Nitrospira* lineage II), and AOB (“GCA-2721545” genus *Nitrosomonadaceae*) (Fig. 3; Fig. S4 and S5). Further details on bacterial nitrifier diversity and distributions can be found in the Supplemental Results, subsection “Bacterial nitrifier MAGs.”

### Osmoregulation genes

Genes associated with different osmoregulation strategies were also present in nitrifier MAGs. Generally, AOA and AOB lacked genes for uptake or synthesis of compatible solutes, although many of these genes were present in NOB and comammox MAGs (Fig. S6). AOA and bacterial nitrifiers generally encoded different sodium/proton antiporters, mechanosensitive channels, and potassium uptake systems (Fig. S6). Few bacterial nitrifiers encoded aquaporins relative to archaea; most AOA had at least one aquaporin gene, and some encoded two (Fig. S6). A more detailed description of osmoregulation genes can be found in the Supplemental Results.

### Dissolved inorganic N uptake and utilization

**Fig 4:**
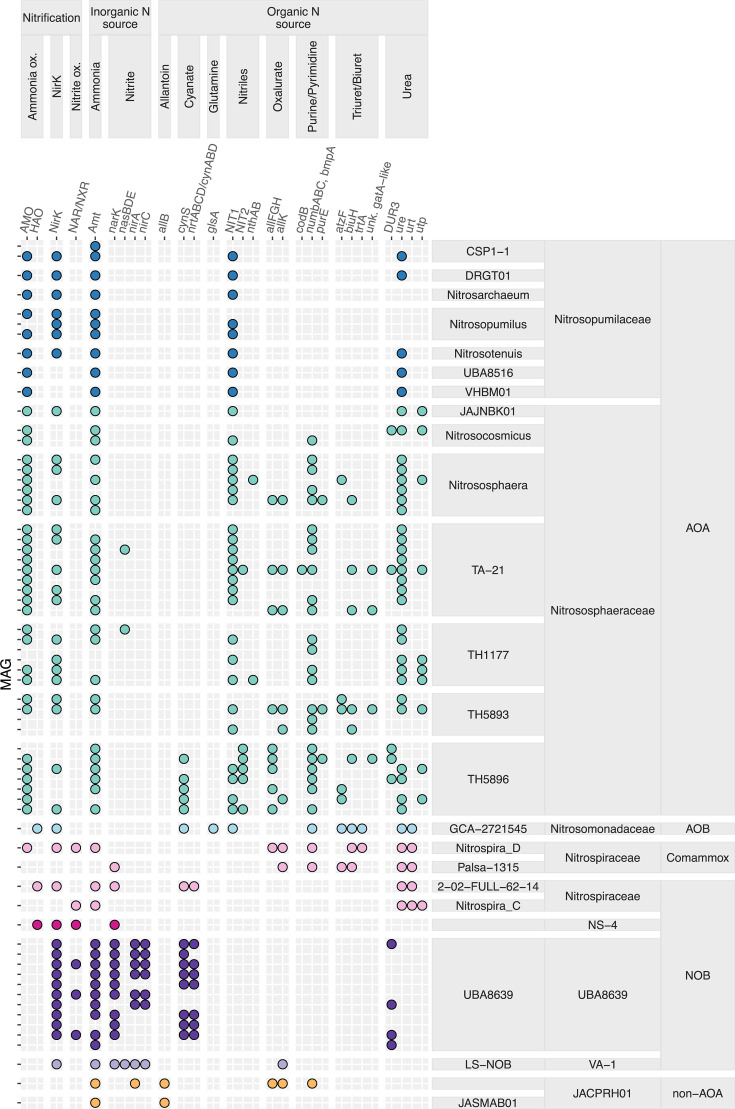
N cycling gene presence-absence in nitrifier lineage representatives. Genes include: ammonia monooxygenase (AMO: *amoABC*), hydroxylamine oxidoreductase (*hao*), nitrate reductase/nitrite oxidoreductase (NXR/NAR: *nxrAB*), nitrite reductase (*nirK*), ammonium transporter (*amt*), nitrite transporter (*nirC*), NNP family nitrate/nitrite transporter (*narK*), nitrite reductase (*nirA, nasBDE*), allantoinase (*allB*), cyanate lyase (*cynS*), nitrite/nitrate/cyanate transporter (*nrtABCD/cynABD*), glutaminase (*glsA*), nitrilase (*nit1*), nitrile hydratase (*nthA*), omega amidase (*nit2*), oxamic transcarbamylase (*allFGH*), carbamate kinase (*allK*), cytosine permease (*codB*), ABC-type nucleoside transporter (*bmpA-nupABC*), 5-carboxyaminoimidazole ribonucleotide mutase (*purE*), allophanate hydrolase (*atzF*), biuret hydrolase (*biuH*), triuret hydrolase (*trtA*), uncharacterized *gatA*-like amidohydrolase, urease (*ure*), urea transport system (*urt*), urea transporter (*utp*), and urea-proton symporter (*dur3*).

Ammonium transporter genes (*amt*) were annotated in most (82%) nitrifier lineages excluding the AOB lineage (Fig. 4; Fig. S7). Over half (59%) of the *amt*-encoding lineages encoded two or more *amt* genes. Based on gene phylogeny, AOA *amt* genes can be classified as encoding either a high-affinity transporter or a low-affinity transporter (43), which we refer to as Amt1 and Amt2, respectively (44–47). A total of 26 AOA lineages encoded the low-affinity *amt1*, and 25 AOA lineages encoded the high-affinity *amt2*, with 16 encoding both (Fig. S8). AOA MAGs harbored the expected ammonia monooxygenase genes (*amoABC*), which were absent from the two non-AOA *Nitrososphaerales* MAGs (Fig. 4; Fig. S8). AOB MAGs contained genes for ammonia oxidation (*amoABC*, *hao*) (Fig. 4; Fig. S8). Of the two proposed comammox lineages present in WRB, *Nitrospira_D* MAGs encoded *amoABC*, *hao*, and *nxrAB*, in contrast to the less complete Palsa-1315 MAG, which lacked these genes (Table S1; Fig. S4). Despite lacking these nitrification genes, the Palsa-1315 MAG was closely related to comammox MAGs from SR and ER (Fig. S4) and harbored genes for utilizing biuret/triuret (Fig. 4, discussed in detail below), supporting our assertion that it represents a comammox lineage. The *nxrAB* genes had a patchy distribution in both established and putative NOB MAGs from WRB and were missing from 67% of representative MAGs (Fig. S8). The nitrite/nitrate transporter (*narK*) was encoded by most (79%) *Nitrospirales* NOB MAGs and the Palsa-1315 MAG. Additionally, the UBA8639 (*Nitrospira* lineage IV) and LS-NOB (*Nitrospinales*) MAGs encoded nitrite transporter, *nirC*. UBA8639 MAGs harbored genes for assimilatory nitrite reduction (*nirA*) as did LS_NOB (*nirA*, *nasBDE*) (Fig. 4; Fig. S8).

### Proposed pathways for utilizing purine degradation products

**Fig 5:**
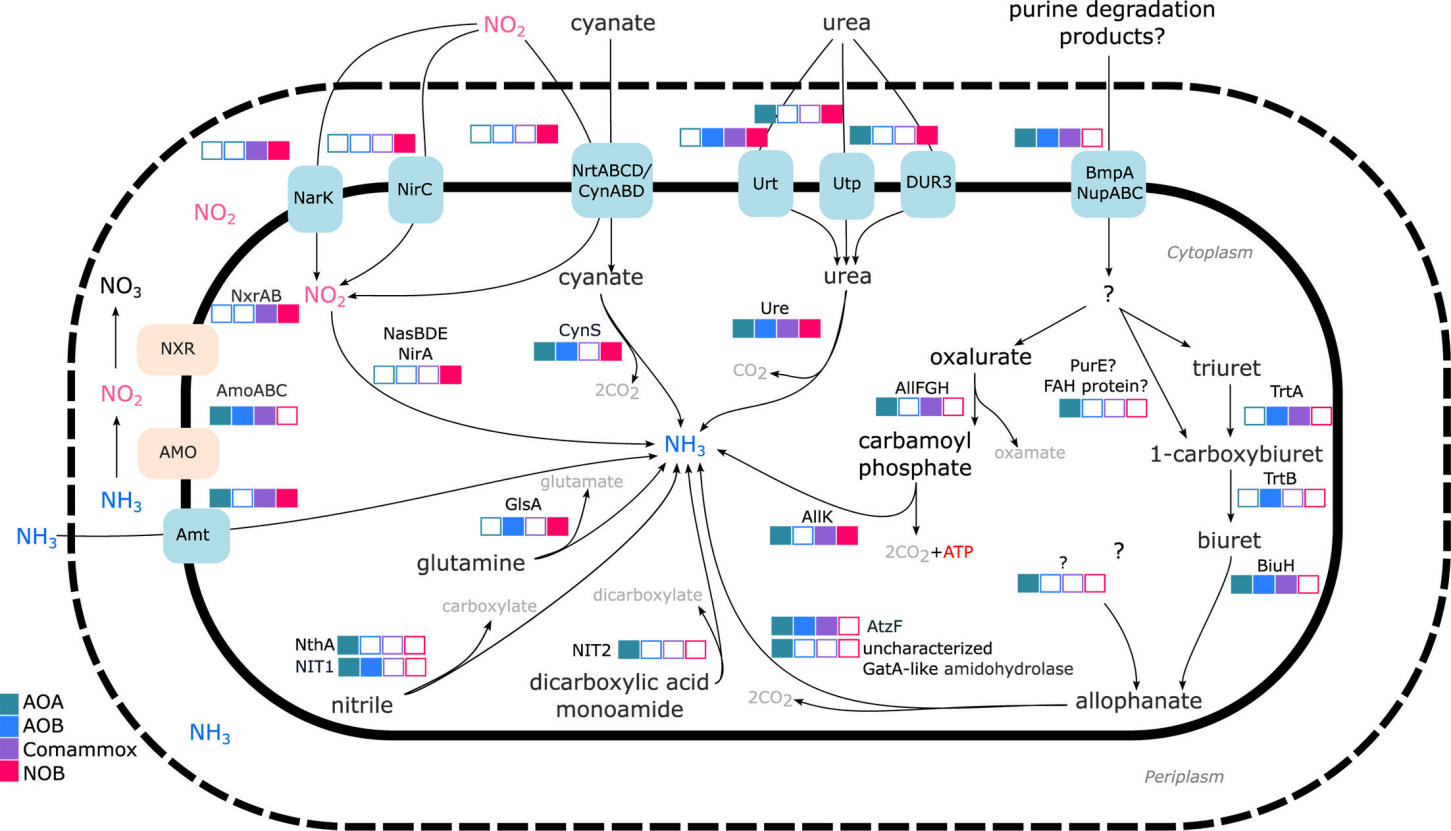
Schematic of predicted N cycling metabolisms in WRB nitrifier MAGs. Filled boxes indicate the presence of a given gene in at least one MAG of a specified nitrifier guild. Select functional genes were used to represent metabolic processes, including ammonium transport (Amt), ammonia oxidation (AmoABC), nitrite oxidation (NxrAB), nitrite transport (NirC), nitrite/nitrate transport (NarK), assimilatory nitrite reduction (NirA, NasBDE), nitrite/nitrate/cyanate transport (NrtABCD/CynABD), cyanate hydrolysis (CynS), urea transport (Urt, Utp, DUR3), urea degradation (Ure), glutamine deamination (GlsA), nitrile degradation (NIT1, NthA), dicarboxylic acid degradation (NIT2), ABC-type nucleoside transport (BmpA-NupABC), triuret hydrolysis (TrtA), carboxybiuret decarboxylation (TrtB), biuret hydrolysis (BiuH), allophanate hydrolysis (AtzF, uncharacterized GatA-like amidohydrolase), oxalurate degradation (AllFGH), and carbamate degradation (AllK). Note that the orientation of AMO has yet to be confirmed.

Urea is a common product of purine and pyrimidine degradation. Most (77%) ammonia oxidizer MAGs along with 40% of NOB lineages from the WRB harbored at least one urease subunit (Fig. 4; Fig. S8). Three types of urea transporters were present and differently distributed across nitrifier guilds (Fig. 4 and 5). The ABC urea transport system (*urtABCDE*) was encoded by both comammox lineages (*Nitrospira_D*, Palsa-1315) and *Nitrospiraceae* NOB (2-02-FULL-62-14, *Nitrospira_C*) (Fig. 4; Fig. S8). A urea transporter (*utp*) was found throughout the *Nitrososphaeraceae* family and in *Nitrospira_C* (Fig. 4; Fig. S8). Lastly, the urea-proton symporter (DUR3) was encoded by a total of five AOA lineages from the *Nitrososphaeraceae* (*Nitrosocosmicus*, *Nitrososphaera*, TA-21, TH5893, TH5896) and *Nitrosopumilaceae* (*Nitrosopumilus*, VHBM01) (Fig. 4; Fig. S8) and four UBA8639 lineages (Fig. S8). Although nine UBA8639 MAGs encoded DUR3 in total, urease or other urea transporter (*utp*, *urt*) genes were completely absent (0/51 MAGs; Fig. S8).

**Fig 6:**
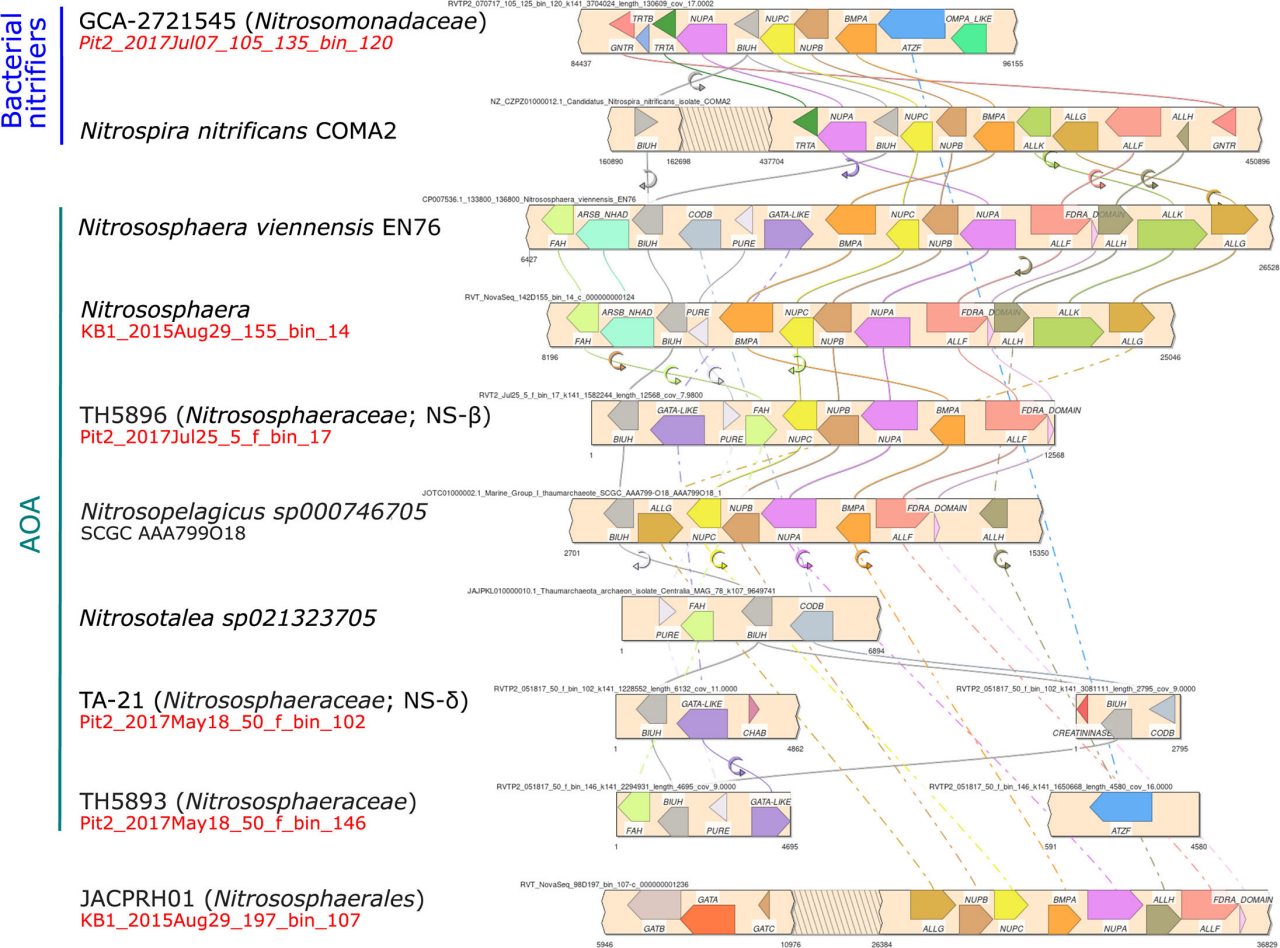
Gene synteny of *biuH*-harboring contigs in bacterial and archaeal ammonia-oxidizer genomes. Annotated genes include allophanate hydrolase (*atzF*), biuret hydrolase (*biuH*), triuret hydrolase (*trtA*), carboxybiuret decarboxylase (*trtB*), ABC-type nucleoside transporter (*bmpA-nupABC*), oxamic transcarbamylase (*allFGH*), catabolic carbamate kinase (*allK*), *gntR* family transcriptional regulator, cytosine permease (*codB*), intermolecular transferase 5-(carboxyamino)imidazole ribonucleotide mutase (*purE*), fumarylacetoacetate hydrolase family protein (FAH), creatininase, and an uncharacterized amidohydrolase (*gatA*-like). Extended data in Fig. S12 and S15. WRB MAG names in red.

Genes related to guanidine degradation (i.e., guanidinase, guanidine carboxylase, carboxyguanidine deiminase, and APC superfamily permease) were absent from all WRB nitrifier MAGs based on a *blastp* search (e-value cutoff 1e-20). However, MAGs from all three ammonia oxidizer guilds encoded genes annotated as allophanate hydrolase (*atzF*) (Fig. 4 and 5). The *atzF* sequences from AOB MAGs did not fall into a clade with the *atzF* of guanidine-degrading AOB and comammox (*Nitrosomonas*, *Nitrosospira*, and *Nitrospira*) but rather were more closely related to *atzF* sequences from other *Nitrosomonadaceae* and *Herbaspirillum* (Fig. S9). In AOB from WRB, *atzF* was collocated with genes encoding biuret hydrolase (*biuH*), a putative triuret hydrolase (*trtA*) based on phylogeny (Fig. S10), a putative carboxybiuret decarboxylase (*trtB*) (Fig. S11), and an ABC nucleoside transporter (*bmpA-nupABC*) (Fig. 6). Generally, triuret degradation requires two isochorismatase-like hydrolases (*trtA*, *biuH*), a decarboxylase (*trtB*), and an amidohydrolase (*atzF*) (31); thus, this contig harbored a complete pathway for triuret degradation. Nearby on the same contig was a complete urease (*ureABCDEFG*) cassette.

Similar to the pathway observed in AOB, *Nitrospira_D* MAGs harbored *atzF* collocated with *biuH*, putative *trtA*, and *bmpA-nupABC* (Fig. S12). Additionally, genes encoding the oxalurate degradation pathway were located next to the triuret degradation pathway, including *allFGH* (formerly *fdrA/yahF*, *ylbE/DUF1116*, and *ylbF/DUF2877* [48]) and carbamate kinase (*allK*, formerly *arcC/ybcF* [49]) (Fig. S12), which are discussed in further detail in the Supplemental Results. Oxalurate degradation is the final step in the oxamate branch for the ALL allantoin degradation pathway (50). The Palsa-1315 MAG from WRB also encoded *biuH* and *atzF*. Known genes for upstream purine degradation were absent from both AOB and comammox MAGs. There was general gene synteny of the triuret degradation pathway between bacterial nitrifiers from the WRB; however, comammox contigs lacked *trtB* and also encoded *allFGHK* (Fig. 6; Fig. S12). There was strong gene synteny between *Nitrospira_D* and Palsa-1315 genomes harboring this pathway, including in the enriched *Ca*. Nitrospira nitrificans COMA2 strain (51) (Fig. S12).

A total of 12 AOA MAGs encoded genes annotated as *atzF*, including MAGs from three distinct genera: *Nitrososphaera*, TH5893, and TH5896. Contigs harboring *atzF* were generally short and encoded genes for uncharacterized and hypothetical proteins (Fig. S13). Additionally, of the WRB AOA encoding *atzF,* only three also encoded *biuH* (Fig. 2 and 4). Only 2 out of 554 GTDB species representatives within the *Nitrososphaeria* and 1 River Thames AOA MAG encoded *atzF* based on a *blastp* search (Fig. 2, e-value cutoff 1e-40). Three WRB AOA harbored multiple copies of *atzF*, sometimes located on the same contig (Fig. S13). The amino acid sequences of AtzF from AOA fell into an archaeal clade most closely related to bacterial AtzF and not other amidohydrolases found in other purine catabolism pathways (Fig. S9). Compared to structurally characterized AtzF from *Pseudomonas* sp. 47660, *Kluyveromyces lactis*, and *Granulibacter bethesdensis*, the AOA AtzF sequences contained several insertions and a truncated C-terminus (Fig. S14A).

**Fig 7:**
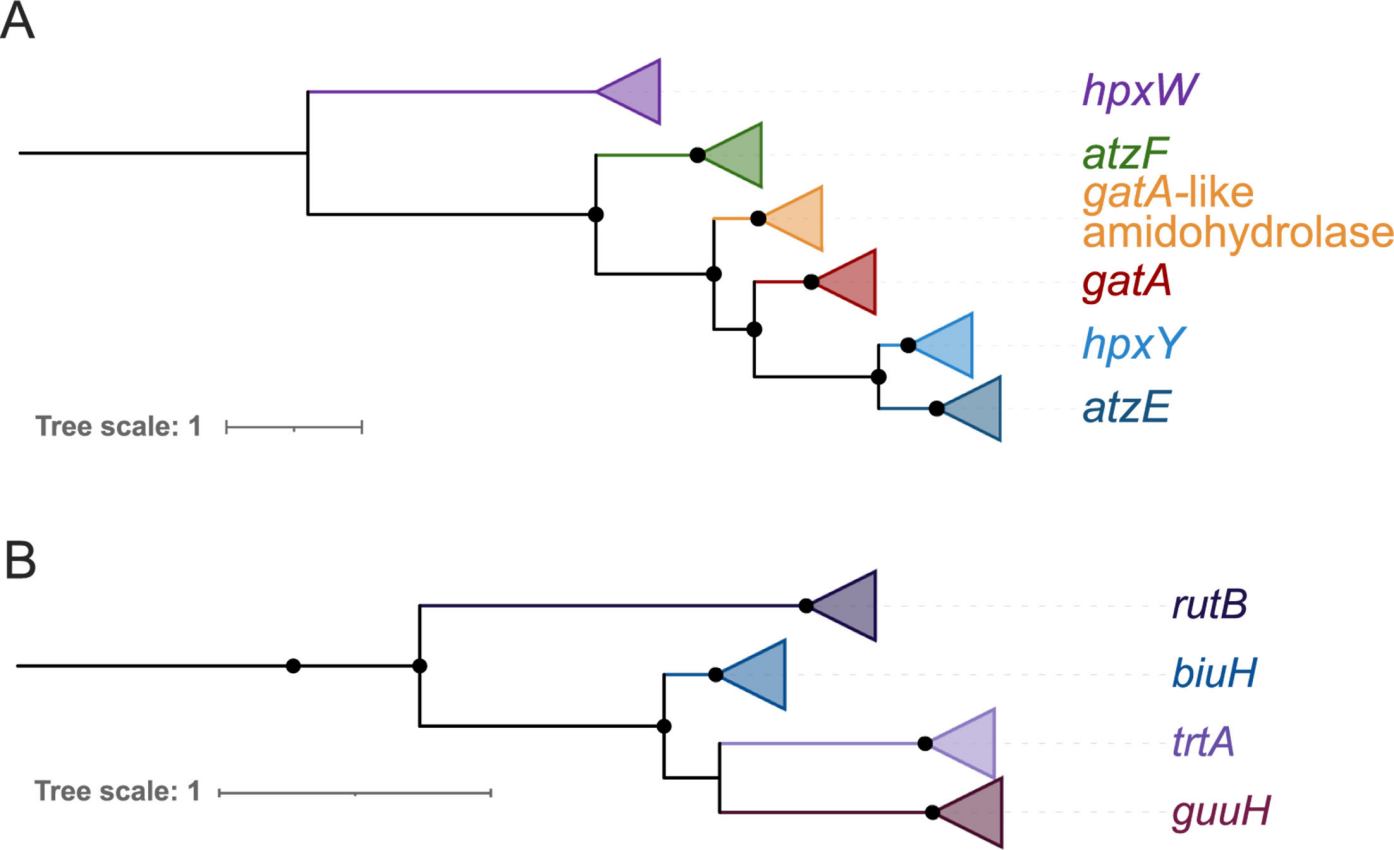
(**A**) Phylogeny of amidohydrolase genes, including allophanate hydrolase (*atzF*, green branches)*,* carboxybiuret hydrolase (*atzE*)*,* glutamyl-tRNA amidotransferase (*gatA*), oxamate carbamoyltransferase (*hpx*Y)*,* oxamate amidohydrolase (*hpxW),* and the uncharacterized *“gatA-like”* amidohydrolase from AOA (orange branches, based on a 533 position amino acid alignment of 150 sequences made with IQ-TREE2 model LG + G4. Extended data in Fig. S9. (**B**) Phylogeny of isochorismatase genes, including biuret hydrolase (*biuH*)*,* triuret hydrolase (*trtA*), guanylurea hydrolase (*guuH*), and ureidoacrylate amidohydrolase (*rutB*), based on a 257 position amino acid alignment of 234 sequences made with IQ-TREE2 model LG + R6. Extended data in Fig. S10. The tree is pruned to exclude distantly related structurally characterized isochorismatase-family proteins. Black dots indicate bootstrap support of ≥ 90%, and trees are midpoint rooted.

In total, 19 AOA MAGs from the WRB encoded genes that were annotated as *biuH* (Fig. 4; Fig. S8). These *biuH* sequences were distinct from other isochorismatase-like hydrolases, such as *trtA* and guanylurea hydrolase (*guuH*) (52) (Fig. 7; Fig. S10), and were in agreement with findings in a previous study identifying a high-confidence *biuH* sequence from *Nitrososphaera* (53). In a midpoint-rooted tree, AOA *biuH* sequences formed a cluster containing archaeal and bacterial *biuH* sequences, including sequences from the AOB *Nitrosospira*, that was sister to a bacterial *biuH* cluster containing sequences from many bacterial genera including *Herbaspirillum*, *Nitrospira*, and members of the *Nitrosomonadaceae* (Fig. S10). Compared to the structurally characterized BiuH enzyme of *Herbaspirillum* sp. strain CAH-3 (54) and *Rhizobium leguminosarum* bv. *viciae* 3841 (55), the AOA sequences had an extended N-terminal and several insertions (Fig. S14B). Like the AOB and comammox contigs that encoded *biuH*, AOA contigs also encoded an ABC nucleoside transporter (*bmpA-nupABC*). In contrast to bacterial ammonia oxidizers, however, AOA did not encode a collocated *atzF* and did not have strong gene synteny across genera (Fig. 6; Fig. S15). Most (68%) *biuH*-encoding AOA MAGs encoded an uncharacterized amidohydrolase annotated as a glutamyl-tRNA amidotransferase subunit A (*gatA*) next to *biuH*. This “*gatA*” diverged from the *gatA* sequence found in *gatCAB* cassette, which encodes the glutamyl-tRNA amidotransferase present in most archaea (56) and found in most AOA MAGs generated in this study. Additionally, “*gatA*” was phylogenetically distinct from closely related amidohydrolases in the ALL and HPX purine catabolism pathways (50), including 1-carboxybiuret hydrolase (*atzE*) and oxalurate amidohydrolase (*hpxY*) (Fig. 7; Fig. S9). Like the comammox *biuH*-harboring contigs, AOA *biuH* contigs also often encoded *allFGHK* for oxalurate degradation (Fig. 6). In several AOA, *biuH* was collocated with a fumarylacetoacetate hydrolase (FAH) family protein gene, which is essential in aromatic degradation pathways (57), and an intermolecular transferase 5-(carboxyamino)imidazole ribonucleotide mutase (*purE*) (Fig. 6; Fig. S15). A few AOA also harbored a cytosine permease (*codB*), and one WRB MAG harbored two *biuH* sequences—one located near a gene annotated as a creatinine amidohydrolase, most closely related to other creatininase-like sequences from uncultured *Nitrososphaeraceae*, but distinct from those found in cultured *Nitrosopumilaceae* and some cultured *Nitrososphaeraceae* creatininase-like sequences (Fig. S16). One of the non-AOA *Nitrososphaerales* MAGs encoded *allFGHK* and *bmpA-nupABC* on the same contig as *gatCAB* (Fig. 6; Fig. S15). No AOA MAGs encoded known genes for upstream purine degradation.

Six non-redundant AOA MAGs encoded *biuH* and were present in 85% of samples from the floodplain at an average abundance of 5.4 RPKG (range = 0.2–101 RPKG) per sample. We also identified *biuH* sequences in 27 genomes out of the 554 GTDB species representatives queried using *blastp* (e-value cutoff 1e-40), including three genomes generated from River Thames sediments, three genomes from the *Nitrosopumilaceae*, and the cultured strain *Nitrososphaera viennensis* EN76 (58) (Fig. 2). A small number of genomes encoded multiple *biuH* sequences that were not identical (Fig. S10).

### Cyanate, nitrile, and glutamine degradation genes

Genes for uptake and utilization of cyanate were found in 24% of WRB nitrifier lineages. About two-thirds of UBA8639 MAGs encoded cyanase (*cynS*) and a nitrite/nitrate/cyanate ABC transporter (*nrtABCD/cynABD*), as did ~80% of 2-02-FULL-62-14 (*Nitrospiraceae*) MAGs. Several AOA (70%) from the TH5896 genus, one TA-21 MAG, and the AOB lineage encoded *cynS* (Fig. S8). The *cynS* sequences from WRB nitrifiers fell into two separate clades; one clade included sequences from many nitrifiers, including *Nitrosospira*, *Nitrosococcus*, *Nitrosomonas*, NOB *Nitrospira*, comammox *Nitrospira*, and *Nitrospina*, as well as anammox, and was related to eukaryotic *cynS*, while the other clade included *Nitrospirales*, *Nitrososphaeria,* methanotrophs, and archaeal sequences (Fig. S17). The *cynS* sequences from UBA8639 (*Nitrospira* lineage IV) MAGs fell into both clades (Fig. S18). The *cynS*-encoding nitrifier lineages were present in 84% of samples at an average total abundance of 17 RPKG per sample.

Regarding the breakdown of nitrile compounds, the gene encoding a putative nitrilase, NIT1, was present in 71% of AOA lineages and in the AOB lineage. Additionally, two AOA lineages—one from *Nitrososphaera* and one from TH1177—encoded nitrile hydratase subunit A (*nthA*), and 1 TA-21 lineage and 5 TH5896 lineages encoded a putative nitrilase/omega-amidase (NIT2). The AOB lineage and 10 MAGs from the numerically dominant NOB genus (UBA8639, *Nitrospira* lineage IV) encoded a glutaminase (*glsA*) that can convert glutamine to glutamate while releasing ammonia (Fig. 4; Fig. S8). The N-cycling gene content of WRB AOB compared to other *Nitrosomonadaceae* is discussed in further detail in the Supplemental Results.

## DISCUSSION

### Diverse and marine-like nitrifiers found in WRB floodplain sediments

The taxonomic diversity and depth-differentiated structure of AOA MAGs recovered in this study are in line with previous *amoA* and 16S rRNA gene amplicon studies at this site near Riverton, WY (11, 12). The AOA genera recovered in this study expand upon the MAGs reported for 2015 samples (13) to include lineages from the GTDB-defined genera UBA8516 (*Nitrosopumilaceae*), TA-21 (*Nitrososphaeraceae*), TH5893 (*Nitrososphaeraceae*), and *Nitrosocosmicus* (*Nitrososphaeraceae*). Many abundant lineages of AOA in the WRB floodplain sediments are related to *Nitrososphaeraceae* MAGs from sediments of the River Thames, including TH5896, TH5893, and TH1177. Phylogenomic reconstruction and analysis of proteome content changes indicate that lineages in the *Nitrososphaeraceae* have undergone many gene duplication and loss events, allowing for distinct and often large proteomes to emerge and for lineages to specialize to different niches selected for by the environment (59). These patterns in genome evolution could explain the prevalence of *Nitrososphaeraceae* in the dynamic WRB floodplain sediments. In addition to the diverse AOA genera, several genera of established and putative NOB from the *Nitrospirales* and *Nitrospinales*, two comammox lineages, and an AOB lineage were recovered from WRB floodplain sediments. The greater taxonomic diversity of nitrifiers and numeric dominance of AOA in the WRB is in contrast to the SR floodplain nitrifier community, which was numerically dominated by comammox bacteria, AOA adapted to oligotrophic conditions, and putative NOB from the NS-4 family (*Nitrospirales*) (10). However, like SR nitrifier MAGs, WRB MAGs encoded the genetic potential to use many different organic N compounds as N and/or energy sources.

Marine and estuarine-like nitrifier lineages were found in deeper depths of WRB floodplain sediments. For example, *Nitrosopumilaceae*, including *Nitrosopumilus* and *Nitrosarchaeum*, were the most abundant AOA in deeper depths of WRB sediments. The NOB lineage, UBA8639 (*Nitrospira* lineage IV), was cosmopolitan in WRB, and LS-NOB (Clade 2 *Nitrospinae*) was found in deeper depths. Both of these NOB groups tend to be associated with marine environments (60–62). The AOB in WRB were also closely related to marine lineages (Fig. S5). This could be due to high sulfate concentrations in groundwater (~4.5–9 g/L) or higher salinity in the transiently saturated and transition zones (~6.5–11.5 ppt) of WRB soils (12, 63, 64). Nitrifier MAGs encoded several different strategies for osmoregulation (compatible solutes versus salt-ion exchange) that varied between different guilds and genera, potentially allowing organisms to adapt to the dynamic floodplain and variable osmotic pressure outside the cell.

### Putative novel pathways indicate additional purine degradation products could support ammonia oxidation

A common degradation product from both purine and pyrimidine catabolism is urea, which has been established as an important energy and N-source for nitrifiers in a range of environments (17, 19, 20, 65). Indeed, urease was encoded by a little over half of the WRB nitrifier MAGs presented in this study and was found across all four nitrifier guilds (28). However, outside of urea, upstream purine degradation products can supply N to microorganisms (66), and this study suggests that they may also support nitrification. Purines can be completely degraded through several different aerobic and anaerobic pathways, generally first metabolized to xanthine, followed by conversion to key intermediates urate and allantoin (50, 66, 67). Allantoin can then be metabolized aerobically through allantoate to glyoxylate and ammonia, or anaerobically through glyoxylate and oxalurate to 2-phosphoglycerate, urea, oxamate, and ammonia (50). Here, we identified downstream purine degradation pathways, such as oxalurate and/or triuret/biuret degradation, in AOA, AOB, and comammox (Fig. 4).

The AOB from the WRB harbored genes comprising a triuret degradation pathway similar to that described in *Herbaspirillum* (31), where triuret is hydrolyzed to carboxybiuret (*trtA*), then decarboxylated to biuret (*trtB*), hydrolyzed to allophanate (*biuH*), and finally hydrolyzed to ammonia (*atzF*) (Fig. 5 and 6). Comammox from WRB (as well as SR) are likely capable of degrading triuret in a similar manner as AOB, though a *trtB* is absent, indicating the carboxybiuret decarboxylation step may be carried out by an unidentified enzyme or happen spontaneously (31). In AOA, the potential pathways for degrading triuret or biuret are less clear (Fig. 6; Fig. S15). Although several AOA from WRB sediments harbored a *biuH* gene, the gene synteny of contigs suggests that multiple possible pathways could occur. Further biochemical studies are necessary to confirm the substrate utilized by the proposed *biuH* in AOA. However, we suggest that the BiuH enzyme likely acts on one or more of the following: triuret, carboxybiuret, and/or biuret. We suggest that the uncharacterized “*gatA*-like” amidohydrolase collocated with *biuH* may function as an allophanate hydrolase, allowing triuret/biuret/carboxybiuret to be degraded through allophanate to ammonia. Some *biuH*-contigs in AOA also encode FAH family proteins that could function in ureide degradation, and the common co-occurrence of *purE* also suggests that some other, potentially upstream purine degradation product may be further degraded by AOA themselves to become triuret/biuret/carboxybiuret. Some *biuH*-contigs also encode a cytosine permease (*codB*), indicating that pyrimidine breakdown products may also be used in these pathways.

Both comammox and AOA encoded *allFGHK* which could allow them to carry out the last steps of allantoin degradation: oxalurate degradation to oxamate and ammonia (50). The collocation of genes for both biuret and oxalurate degradation pathways in AOA and comammox suggests that these ammonia oxidizers may utilize several different purine degradation products produced by other microbial community members, as the upstream genes for allantoin/purine degradation were absent from nitrifier MAGs. It has recently been proposed that purine and pyrimidine cross-feeding is common in the global oceans between abundant community members SAR11, *Prochlorococcus*, and AOA (23); perhaps purine metabolic handoffs (68) are occurring in the WRB.

Despite metabolic versatility to use several organic N sources, nitrifiers in WRB lack the genetic potential to degrade guanidine, as recently described for some comammox and potentially AOB (29). However, several AOA harbor *atzF* functioning in an unknown pathway. It is possible that AOA produce allophanate from some purine- or pyrimidine-related degradation process; alternatively, allophanate could be produced by other community members and then taken up and degraded by AOA. The *atzF* gene appears to be rare in AOA genomes overall (Fig. 2), and physiological and biochemical studies are required to confirm whether *atzF* in AOA acts on allophanate as a substrate.

### Dynamic floodplain environment may select for nitrifiers utilizing organic N compounds

Although aerobic ammonia oxidizers generally have high substrate affinity for ammonia, this affinity varies between different guilds (i.e., AOB versus AOA versus comammox [69, 70]) and lineages (i.e., marine versus terrestrial AOA [71]). This variability in substrate affinity is thought to play a key role in the competition between and niche partitioning of AOA, AOB, and comammox in the environment (72–76). Not only do aerobic ammonia oxidizer guilds have different affinities for ammonia but also different preferences for utilizing ammonia versus other N substrates when available. In culture-based experiments, both AOA and comammox preferred to utilize ammonia over urea and switched to urea utilization only when ammonia was depleted (27). In contrast, cultured AOB from the *Betaproteobacteria* (i.e., *Nitrosomonas* and *Nitrosospira*) preferentially utilized urea over ammonia, and AOB from the *Gammaproteobacteria* (i.e., *Nitrosococcus*) utilized both urea and ammonia when both substrates were available (27). Preferences for different N sources when multiple are available could allow for co-existence of nitrifiers in different conditions. For example, urea fertilization allows for the co-occurrence of comammox, AOB, and NOB in continuous flow reactors (25), as well as AOA and AOB in soil microcosms (26) and subsurface aquifer sediments (77). In addition to urea, the availability of the substrate cyanate could also impact nitrifier competition, co-occurrence, and niche partitioning. Cyanate can serve as the sole energy and N source for *Nitrososphaera gargensis* in culture (18) and can be used for reciprocal feeding between AOA and NOB (17, 18). How substrate preferences for urea, cyanate, or other organic N compounds impact nitrifier activity and niche partitioning in highly fluctuating environments has yet to be fully explored.

In this study, most nitrifiers in the non-redundant MAG data set (59%) encoded urea transport and urease genes. Not only does urea appear to be of potential importance to all four guilds of nitrifiers, but *cynS* was also encoded in many (24%) of these MAGs, including in comammox, NOB, and AOA. Cyanase is not commonly found in AOA genomes (47) and has been reported in *Nitrososphaera garagensis* (18) and previously generated MAGs from the WRB (13); however, cyanate can be both an energy and nitrogen source for AOA in ocean waters of the Gulf of Mexico and *Nitrosopumilus maritimus* cultures, despite the absence of canonical *cynS* (17). In addition to cyanate, the presence of putative *nit1*, *nit2*, and *nthA* in AOA and *nit1* in AOB supports the idea that some nitriles or dicarboxylic acid monamides may also support ammonia oxidation in the WRB. However, the substrates used by these enzymes have not been experimentally verified in nitrifiers, nor has their role in supplying N been confirmed. And as discussed in detail above, proposed degradation pathways for triuret, biuret, oxalurate, and allophanate were present in ammonia oxidizer MAGs in the WRB. The robust presence of genes encoding for organic N utilization raises several questions, including: why might these genes be prevalent in WRB MAGs, and what are the potential sources of organic N in the WRB floodplain?

Floodplains can receive nutrients from multiple sources, including from pulses of stream-derived allochthonous material (e.g., sediments, organic matter), atmospheric deposition, autochthonous organic matter (e.g., leaf litter), and groundwater (78, 79), which is influenced by subsurface exchanges between terrestrial and riverine systems, precipitation, and anthropogenic inputs. Generally, floodplain flow and flood pulses (80) stimulate the release of nutrients from decaying organic matter and previously deposited sediments (81–83). After the snowmelt-driven flow/flood pulse in spring, the WRB experiences summer drought conditions (12) that could decrease the rates of ammonification (39, 84). Although ammonia measurements from the WRB are lacking, the numeric predominance of AOA suggests a low ammonia supply in the floodplain. If ammonification from other microbial community members decreases under dry conditions, nitrifiers may directly degrade organic N compounds to supply ammonia. Net N-mineralization rates can remain unchanged despite differences in ammonification and nitrification rates in floodplain environments (84), perhaps related to nitrifier degradation of organic N compounds.

The WRB floodplain is densely vegetated with steppe flora, such as sagebrush, grasses, and willows, which may act as key sources of organic N outside of the rapid release of nutrients during flooding. Snowmelt-driven inundation, strong evapotranspiration (85), and high sulfate concentrations could all lead to plant stress and production of organic N compounds, particularly allantoin, that supply N to the sediment microbial community. Allantoin is an abundant N compound in plants that accumulates during drought and salinity stress (86, 87) and can also serve as a vital and sole N source for microorganisms (50, 88, 89). Allantoin can be catabolized via several different pathways that generate not only urea but also oxalurate (50). It is possible that plants also generate triuret as a side product during stress and defense responses that produce both uric acid and reactive oxygen species (31–33, 88). Additionally, nitriles may be produced by plants as cyanogenic glycosides and glucosinolates during pathogen defense (64).

In addition to live plants, buried organic matter or microbially produced dissolved organic matter (DOM) could supply organic N. For example, as key components of DNA, RNA, and some coenzymes, purines are released as organic matter is remineralized or during microbial cell lysis. Purines can also be excreted by active microbial communities (90, 91). Additionally, cyanate is found in low abundance but is rapidly cycled in terrestrial environments (92), perhaps originating from organic matter degradation as observed in the ocean. In estuarine and marine environments, a major proposed cyanate source is the photochemical degradation of DOM (93–95), specifically polypeptides and aromatic compounds (95). Cyanate can also form from the non-enzymatic decomposition of the nucleotide precursor/degradation product carbamoyl phosphate (96, 97).

In the WRB, organic N could also originate from upstream or neighboring agricultural activities and include sources, such as herbicides, livestock manure, and fertilizers. Symmetrical (s)-triazines are a suite of anthropogenic compounds, including herbicides, that generally break down to cyanuric acid which can then be degraded into biuret and allophanate (53, 54, 98). Although we did not find cyanuric acid hydrolase (*atzD*) genes in any WRB nitrifier MAGs, other microbes could supply biuret from this pathway to them. Nearby or past livestock grazing could be a source of purines and pyrimidines in floodplain sediments. Livestock manure and fertilizer runoff are common sources of organic N in freshwater systems (99). Decomposing manure produces purines and pyrimidines, impacting microbial carbon and N-cycling in soils and sediments (100–103). Urea-based fertilizer can supply not only urea but also triuret and biuret, which are by-products of urea pyrolysis, and even cyanate, as it can form from the spontaneous urea dissociation in an aqueous solution (104, 105). Nitriles in the terrestrial environment can also come from anthropogenic sources, such as from acrylamide, pharmaceuticals, herbicides, mining, metallurgical processes, wastewater discharge, and fuel combustion (106). Our WRB site is near some human development (Riverton, WY) and is a DOE legacy site due to persistent groundwater contamination from past uranium and vanadium ore processing (107), making other anthropogenic sources of nitriles possible. The presence of human development has shown a clear impact on DOM composition in rivers (108); thus, it is possible that several different anthropogenically-derived organic N compounds could be present in the river and groundwater. Further research could elucidate whether such compounds are present and whether they correlate with the organic N utilization genes described here.

It has been hypothesized that the introduction of s-triazine herbicides has led to the evolution of the enzyme 1-carboxybiuret hydrolase *atzEG* from the *gatA* and *gatC* of the glutamyl-tRNA amidotransferase and that indirect tRNA aminoacylation can be a source of alternative functions (109). The uncharacterized *gatA*-like amidohydrolase found collocated with *biuH* in some AOA MAGs may be an example of an alternative function originating from genes involved in tRNA aminoacylations. In further support of this, these uncharacterized *gatA*-like amidohydrolases are present in lineages of the *Nitrososphaeraceae* (Fig. 4), which phylogenomic analysis and ancestral state reconstruction suggest has undergone a high level of proteome expansion through gene duplication and loss (59). The origination of novel functions could enable some *Nitrososphaeraceae* lineages found in the WRB to utilize additional organic compounds as N sources and thrive in dynamic, and perhaps contaminated, floodplain environments. Physiological studies are necessary to confirm the substrates utilized in the proposed purine degradation pathways described in this study, as well as putative nitrilases/omega amidases annotated in nitrifier MAGs. Additionally, metatranscriptomic or metaproteomic studies would illuminate if and under what conditions the proposed pathways for utilizing organic N are active, and whether nitrifier lineages utilize different substrates *in situ*. The presence of putative novel pathways related to purine degradation, as well as for cyanate and nitrile degradation, in nitrifier genomes suggests both the presence of diverse organic N compounds in WRB sediments that warrant characterization and a potentially large role for nitrifiers in net N mineralization.

### Conclusions

Diverse AOA MAGs were recovered from WRB floodplain sediments. Our findings add additional AOA lineages to those reported in Reji et al. (13), as well as highlight the diversity and genetic potential of AOB, comammox, and NOB MAGs present in floodplain sediments. We observed taxonomically diverse and depth-differentiated nitrifier populations with marine-like lineages residing in deeper depths. Several different mechanisms for utilizing organic N sources are present in MAGs and include genes related to utilizing cyanate, nitriles, and glutamine, as well as purine degradation products, such as urea, triuret, biuret, oxalurate, and allophanate. This study increases our understanding of the possible N substrates supporting nitrification and suggests that dynamic floodplains can select for metabolically versatile nitrifiers. Given the presence of *biuH* and *allFGHK* in a cultured representative of AOA (*Nitrososphaera viennensis* [58]) and an enriched comammox (*Ca*. Nitrospira nitrificans [51]), future laboratory studies utilizing different substrates to supply N would yield great insights into the proposed pathways and substrates used by the enzymes identified in this study.

## MATERIALS AND METHODS

### Field sample collection

Floodplain sediments in the WRB were sampled for microbial community analysis near Riverton, WY, USA, at three nearby sites across three field seasons (2015, 2017, and 2019) (Fig. 1). In 2015, samples were collected in August at site KB1 (42.98871, −108.39963) from 0 to 234 cm below ground surface (detailed sampling methods and sediment characteristics available in [11, 13]). In 2017, samples were collected in May, twice in July, and September at site Pit2 (42.98864, −108.40006) from 10 to ~150 cm (detailed sampling methods and sediment characteristics available in [12] and associated geochemical data in [110]). In 2019, samples were collected at site PTT1 (42.98831, −108.40036) in June, August, and October from 60 to 180 cm. All three sites are located on an alluvial terrace near the confluence of the Wind River and Little Wind River with varying distance (50 to 100 m) from the Little Wind River main channel (Fig. 1). Sampling depths include dry, unsaturated sediments to transiently wet and saturated depths (Fig. 1). In general, sediment samples for microbial analyses were collected at discrete depths at ~10–30 cm intervals (depending on soil horizonation, sampling year, and location) along the length of the core, flash-frozen in liquid nitrogen or on dry ice, and stored at −80°C until nucleic acid extraction.

### Metagenome and MAG processing

DNA extraction methods are available in the Supplemental Materials and Methods. A total of 68 DNA samples were submitted to and sequenced by the DOE Joint Genome Institute (JGI) either via a FICUS project (Proposal ID: 504298) or a CSP Project (Proposal ID: 1927). Quality-controlled filtered raw metagenome data (accessions available in Table S2) were downloaded from the JGI Genome Portal for assembly, binning, and refining using the metaWRAP (v1.3.2) pipeline (111). Assemblies were made using MEGAHIT (v1.3.3). Samples from 2019 were also co-assembled by depth. All assemblies were binned using contigs >2,000 nt with MetaBAT2 (v2.12.1) (112) and MaxBin 2.0 (v2.2.6) (113). Single sample assemblies from 2017 were also binned using multiple fastq files and additionally using CONCOCT (v1.0.0) (114). All bins generated from a given metagenome were consolidated and filtered using *metawrap bin_refinement* and then further refined with MAGpurify (v1.0) (115). Any MAGs with >50% completeness and >10% redundancy were manually refined using anvi’o (v8) (116) *anvi-refine* and *anvi-summarize*. MAGs were retained if they were determined to be above >50% completeness and have <10% contamination as calculated via CheckM (v1.1.3) (117). All MAGs were dereplicated using dRep (v3.1.1) (118) at 98% ANI to create a non-redundant MAG data set, and we refer to these representative MAGs as lineages. Taxonomic classification for each representative MAG was made using the GTDB toolkit (GTDB-tk, v2.4.0) (119) with the database release RS220. Reads were competitively recruited to non-redundant MAG data set using Bowtie2 (v2.4.2) (120) with default parameters. Coverage values and read alignment rates per genome were calculated using CoverM (v0.4.0) (121). Abundances are displayed as unpaired reads recruited per genome size of MAG in kilobases of MAG, divided by gigabases of metagenome (RPKG). Genes were called and translated using Prodigal (v2.6.3), and annotations were performed via KEGG (122, 123) using GhostKOALA (v3.1) (124) and eggnog-mapper (v2.1.12) with the eggnog 5 database (125, 126). Code used in this workflow is available on GitHub (https://github.com/anna-rasmussen/MAG-analysis).

### Phylogenetic analysis

Protein-protein BLAST (*blastp*) was used to query WRB MAGs and GTDB species representative genomes for genes of interest, or genes were selected from WRB and SR MAGs based on KEGG (122, 123) annotation. SR nitrifier MAGs were downloaded from PRJNA1142551 and ER from reference 127. Reference protein sequences were identified via *blastp* searches against the PDB, uniprot, refseq select, and nr databases using default parameters or by searching by gene name in PDB. Gene synteny of *biuH* and *atzF* contigs across different genomes was generated and visualized using SimpleSynteny (v1.6b) (128) with a significance value of 0.001 and 50% alignment length. If multiple genes were mapped to a given gene, the one with the highest bit score was kept. Gene sequence alignments were carried out using Clustal Omega (v1.2.3) (129), and the ends were manually trimmed in Geneious Prime (v2024.0.7). Concatenated ribosomal alignments were created using anvi’o with *anvi-get-sequences-for-hmm-hits*, aligned using MUSCLE (v3.8.1551) (130), and trimmed using trimAl (v1.4.rev15). All phylogenetic trees were constructed using IQ-TREE2 (v2.0.3) (131) with ModelFinder *-MFP* (132) to identify the best fit model and 1,000 bootstraps. Phylogenetic trees were visualized using FigTree (v1.4.4) (133) and iTOL (v6) (134). Nucleotide AOA *amoA* sequences were aligned with the updated alignment in (45) based on the unified definitions established in Alves et al. (42) for classification.

## Data Availability

The 68 metagenomes used in this study are available in both NCBI and IMG (JGI) databases, with accession numbers provided in Table S2. All metagenomes can be found under GOLD study IDs Gs0142591 (2017) and Gs0131241 (2015, 2019). All MAGs generated in this study along with quality metrics, GTDB taxonomy, and metagenome of origin accessions are available in the ESS-DIVE data repository under doi:10.15485/2563565 (135), doi:10.15485/2563574 (136), and doi:10.15485/2563566 (137). Nitrifier MAG quality is also available in Table S1.

## SUPPLEMENTAL MATERIAL

Supplemental file msystems.00829-25-s0001.pdf Supplemental text and Fig. S1-S21.

Table S1 msystems.00829-25-s0002.csv GTDB RS220 taxonomy, MAG quality metrics, and metagenome accession numbers for nitrifier MAGs.

Table S2 msystems.00829-25-s0003.csv Metagenome accession numbers for floodplain sediment samples assembled and binned.

ASM does not own the copyrights to Supplemental Material that may be linked to, or accessed through, an article. The authors have granted ASM a non-exclusive, world-wide license to publish the Supplemental Material files. Please contact the corresponding author directly for reuse.
