# Supplementary material for: Floodplain nitrifiers harbor the genetic potential for utilizing a wide range of organic nitrogen compounds
Source: mSystems. 2025 Oct 13;10(11):e00829-25. doi: 10.1128/msystems.00829-25 (PMC12625717; doi:10.1128/msystems.00829-25)
Supplement: Supplemental file — Supplemental text and Fig. S1-S21. [file msystems.00829-25-s0001.pdf]

Supplemental Information for:

Floodplain nitrifiers harbor the genetic potential for utilizing a wide range of organic nitrogen compounds

Anna N. Rasmussen[a], Katie Langenfeld[b§], Bradley B. Tolar[b]*, Zach Perzan[b†], Kate Maher[b], Emily L. Cardarelli[b‡], John R. Bargar[c], Kristin Boye[a], and Christopher A. Francis[b,d]#

SUPPLEMENTAL RESULTS

Bacterial nitrifier MAGs

The 9 *Nitrospinales* MAGs fell within the "LS-NOB" genus and represented 1 NOB lineage after dereplication. The LS-NOB were present in deeper depths (> 140 cm at KB1, > 70 cm Pit2, > 80 cm at PTT1) and at lower abundance than the *Nitrospirales* (Fig. 2). A total of 65 MAGs were generated from the *Nitrospirales* order, including the *Nitrospiraceae* (which includes *Nitrospira* lineages I, II, V, VI, VII), NS-4, and "UBA8639" (*Nitrospira* lineage IV) families.

The vast majority of NOB (n = 51) originated from the Genome Taxonomy Database (GTDB)-defined genus UBA8639 in the UBA8639 family (*Nitrospira* lineage IV). The UBA8639 genus was represented by 11 lineages in WRB sediments and MAGs ranged from medium- to high-quality (Table S1). UBA8639 was present at most depths throughout the floodplain sediment column, however, the different lineages had different depth distributions, and one lineage was absent from KB1 (Fig. S3). From the *Nitrospiraceae*, we recovered MAGs from established NOB, putative NOB, and comammox lineages. One medium-quality MAG (completeness = 74.9%, contamination = 5.4%) was recovered from established NOB genus *Nitrospira_C* (*Nitrospira*

lineage II) and was present in the shallowest sediments in KB1 and Pit2 (surface and 10 cm, respectively) and only at 70 cm in October 2019 at PTT1 (Fig. 3). Also from the *Nitrospiraceae* family, 6 medium- to high-quality MAGs were recovered from the uncultured genus “2-02-FULL-62-14” representing 1 putative NOB lineage (Table S1). These MAGs were closely related to MAGs generated from other riparian floodplains, including Slate River (SR) (1) and East River (ER) (2) (Fig. S4). 2-02-FULL-62-14 were the most abundant NOB in the deepest depths at KB1 (> 140 cm) and were present at low abundance in deeper sediments at Pit2 and PTT1 (Fig. 3). From the uncultured NS-4 family (*Nitrospirales*), 1 medium-quality MAG (completeness = 65.2%, contamination = 1.8%) was recovered representing the same lineage of putative NOB found in floodplain sediments from SR (Fig. S4) (1). The uncultured NS-4 (*Nitrospirales*) lineage was of low abundance but present at all 3 sites in the deepest depths sampled (Fig. 3).

We recovered 5 medium-quality *Nitrospira_D* MAGs and 1 medium-quality MAG (completion = 54.9%, contamination = 4.3%) from the GTDB defined genus “Palsa-1315” (Table S1), both genera that harbor comammox organisms. In WRB, *Nitrospira_D* was represented by 1 MAG after dereplication that encoded ammonia and nitrite oxidation genes (Fig. 4). *Nitrospira_D* comammox (clade A) was present in deeper depths at KB1 (> 100 cm), but absent from Pit2 and rarely present at PTT1 (Fig. 3). Although the Palsa-1315 genus harbors both comammox (clade B) organisms and NOB, the MAG generated from WRB lacked genes encoding both ammonia monooxygenase (AMO) and nitrite oxidoreductase (NXR) (Fig. 4). The Palsa-1315 lineage was present at each site but at low abundance in only the deepest depths sampled (Fig. 3).

The AOB MAGs were of medium- to high-quality (Table S1) and represented only one lineage after dereplication at 98%. The representative AOB MAG was high-quality (completeness = 96.9%, contamination = 0.79%) (Table S1) and was classified as GTDB-defined genus "GCA-2721545" within the *Nitrosomonadaceae* family. GCA-2721545 (*Nitrosomonadaceae)* was sister to *Nitrosomonas* and genomes from this genus in the GTDB originate from marine waters or sediments (Fig. S5). AOB was present in sediments 70-140 cm deep at Pit2 and PTT1, but absent from KB1 (Fig. 3).

***Nitrosomonadaceae* gene content**

In GTDB RS220 there are 5 genera within the *Nitrosomonadaceae*, including established ammonia-oxidizing genera *Nitrosomonas* and *Nitrosospira*. Genomes throughout all 5 genera encoded ammonia monooxygenase and hydroxylamine oxidoreductase (Fig. S5). MAGs from Slate River all represented the same lineage (after dereplication at 98% ANI) of GCA-2721545 and harbored ammonia oxidation machinery supporting that it was an AOB. Several genomes from the GCA-2721545 genera, including the MAGs from SR, encoded *atzF*, *biuH*, *cynS*, and the *urt* instead of the *utp* for urea transport. The *cynS* gene was rare and only present in the GTDB species representatives of GCA-2721545 and not other genera. The *biuH* gene was encoded by members of the GCA-2721545 and *Nitrosospira* genera in contrast to *atzF* which was much more broadly distributed across the *Nitrosomonadaceae* family (Fig. S5). The AOB MAGs from the SR encoded *glsA* which was rarely encoded in other *Nitrosomonadaceae* (Fig. S5). The *Nitrosomonas* sp021774265 genome encoded a gene annotated as *biuH*, however, it fell within the guanylurea hydrolase (*guuH*) clade (Fig. S10) and was colocated with guanidine degradation

genes (Fig. S12). Some *biuH* sequences from *Nitrosospira* were also colocated with guanidine degradation genes (Fig. S12), however, *Nitrosospira* sequences fell within the *biuH* clade and were more closely related to AOA *biuH* than to other bacterial ammonia oxidizer *biuH* sequences (Fig. S10).

Osmoregulation genes

Microorganisms can adapt to osmotic stress through several strategies, for example under hyperosmotic stress microbes can use salt-ion exchange to uptake salts such as potassium commonly referred to as the "salt-in" strategy, or uptake and synthesize compatible solutes, commonly referred to as the "salt-out" strategy (3–6). On the other hand, under hypoosmotic stress mechanosensitive channels can open to allow nonspecific solute efflux during increased osmotic pressure from the cytoplasm and aquaporins can allow for the rapid movement of water across the membrane and fast equilibration (7). AOA MAGs from the WRB mostly lacked genes for uptake or synthesis of compatible solutes such as betaine, ectoine, glycine betaine, proline, or trehalose, with the exception of 1 TH5893 lineage that encoded a glycine betaine transport system (*proVWX*), 2 *Nitrosopumilus* MAGs that encoded ectoine synthase (*ectCD*), and 3 *Nitrosopumilus* MAGs that encoded a betaine/carnitine transporter (Fig. S6). AOA appear to use salt-ion exchange and encoded sodium-proton antiporters including a putative high affinity (*napA*/*nhaS3*-like) and low affinity (*nhaP*/*nhaS1*-like) antiporter (8), mechanosensitive channels such as *kefA* (*Nitrosopumilaceae*), *mscL* (*Nitrosopumilaceae and Nitrososphaeraceae*), and *mscS* (*Nitrososphaeraceae*), potassium uptake mechanisms such as the Trk/Ktr system (*Nitrosopumilaceae and Nitrososphaeraceae*), voltage-gated potassium channel (*kch*)

(*Nitrososphaeraceae*), and potassium-transporting ATPase (*kdpABC*) (*Nitrososphaeraceae*), and aquaporins 4 (*aqp4*) and Z (*aqpZ*) (Fig. S6). Like AOA, AOB also encoded genes only for salt-ion exchange methods including *nhaP/nhaS1*-like sodium-proton antiporter, several mechanosensitive channels (*kefA*, *mscL*, *mscM*, and *ybiO*), potassium uptake (*kch*, *trkHA*), and osmotically inducible protein Y (*osmY*) which has recently been shown to interact with aquaporin Z (9) though these MAGs lack *aqpZ* (Fig. S6). In contrast to AOA and AOB, NOB and comammox MAGs encoded genes for using compatible solutes and different salt-ion exchange mechanisms. Several NOB within the *Nitrospirales* encoded osmoprotectant transport genes (*opuABCD*) for uptake of compatible solutes. Members of the *Nitrospiraceae* and NS-4 encoded genes and for trehalose synthesis (*otsAB*, *treS*, *treYZ*,) and uptake (*thuEFG*) while the most abundant NOB group, UBA8639, encoded only *treS* and *treYZ* for trehalose synthesis (Fig. S6). LS-NOB encoded for ectoine synthase (*ectCD*) (Fig. S6). UBA8639, LS-NOB, and *Nitrospira*_D encoded an Mhn-type sodium-potassium antiporter and UBA8639 also encoded an NhaAB-type (Fig. S6). TheTrk/Ktr system for potassium uptake was missing from the *Nitrospiraceae* NOB but found in other NOB (Fig. S6). The potassium-transporting ATPase (*kdpABC*) was found in comammox MAGs (Fig. S6). Aquaporins were rarely encoded by NOB but many encoded *osmY*, perhaps due in part to the incomplete nature of MAGs (Fig. S6).

Oxalurate catabolism genes

Genes encoding FdrA protein (*fdrA*) and carbamate kinase (*arcC*) were collocated with *biuH* genes in both AOA and comammox MAGs (Fig. 6). These two genes have been renamed to *allF* (10) (Fig. S18) and *allK* (11) (Fig. S19), respectively, and are part of the oxalurate catabolism

pathway. Other nearby genes blasted to DUF1116 domain-containing protein which corresponds to *allG* (formerly *ylbE*) (Fig. S20) and DUF2877 domain-containing protein which corresponds to *allH* (formerly *ylbF*) (Fig. S21). The *allFGH* operon encodes an oxamic transcarboxylase and converts oxalurate to carbamoyl phosphate and oxamate and then *allK* further degrades carbamoyl phosphate to ammonia while producing ATP. In *Escherichia coli*, the *allFGHK* operon is found downstream of other allantoin degradation genes and near *purK* and *purE* (10).

SUPPLEMENTAL MATERIALS AND METHODS

Field site description

The Wind River Basin (WRB), near Riverton, WY, experiences large, snowmelt-driven seasonal flooding that leads to alternating redox conditions depending on water table height and the attendant biogeochemical transformations. Legacy uranium mining has led to uranium and evaporite mineral accumulation in the sediments, as well as groundwater contamination, including high sulfate and ionic strength. A large flooding event occurred in summer of 2016 between sampling years that had significant impacts on the floodplain (12). Site KB1 was sampled in late August 2015 and located closest to the main Little Wind River channel (50 m) next to a small side channel leading to more river water influence than at the other 2 sites. Pit2 was located 80 m from the main Little Wind River channel and sampled in 2017 before (May) and after (July, September) major flood inundation in June. PTT1 was located 100 m from the

main Little Wind River channel and sampled in 2019 4 days after flood inundation (June), during peak evapotranspiration (August), and at plant senescence (October).

DNA extraction

For 2015 samples, DNA was extracted from ~0.3 g of sediment using the PowerSoil DNA Extraction Kit (MoBio, Carlsbad, CA). For 2017 samples, DNA was extracted from 0.25-0.5 g of sediment using the PowerSoil DNeasy Extraction Kit (Qiagen). Extractions followed the manufacturer's instructions after mechanical agitation in a FastPrep bead beater (MP Biomedicals, Santa Ana, CA) for 2 cycles of 30 s at setting 5.5 with a 1 min incubation on ice in between. DNA was eluted after a 1 min incubation at room temperature. For 2019 samples, DNA was extracted from 0.25-0.5 g of sediment using the DNeasy PowerSoil Pro Kit (Qiagen) following manufacturer's instructions (including a 10 min bead beating via vortex with a horizontal tube adaptor) with an additional incubation step at 80 °C for 40 minutes immediately following bead beating to allow for adequate recovery from floodplain sediment as compared to the original PowerSoil Kit formulation. In some cases, multiple extracts from the same sample were pooled at the spin column step to get adequate DNA recovery from low-biomass soils.

Community analysis

Alpha diversity was calculated and ordinations were made in R using *phyloseq* (13) and *vegan* (14) and visualized using *ggplot2*. Only nitrifier MAGs with a coverage fraction >= 0.4 as calculated by coverM were considered "present" in a sample and used in alpha and beta

diversity calculations. Bray-Curtis dissimilarity of MAG abundance was the distance used for both Principal Coordinate Analysis (PCoA) and Constrained Analysis of Principal Coordinates (CAP). Scree plots were visualized using *plot_scree*. The significance of CAP ordination variables ("terms") and axes was assessed using *anova.cca* and step = 1000 with the default α value = 0.05.

REFERENCES

1. Rasmussen AN, Tolar BB, Bargar JR, Boye K, Francis CA. 2024. Metagenome-assembled genomes for oligotrophic, non-canonical nitrifiers recovered from a mountainous gravelbed floodplain harbor the genetic potential for utilizing alternative sources of ammonia. In preparation.

2. Matheus Carnevali PB, Lavy A, Thomas AD, Crits-Christoph A, Diamond S, Méheust R, Olm MR, Sharrar A, Lei S, Dong W, Falco N, Bouskill N, Newcomer ME, Nico P, Wainwright H, Dwivedi D, Williams KH, Hubbard S, Banfield JF. 2021. Meanders as a scaling motif for understanding of floodplain soil microbiome and biogeochemical potential at the watershed scale. Microbiome 9:121.

3. Kunte HJ, Trüper HG, Stan-Lotter H. 2002. Halophilic Microorganisms, p. 185–200. *In* Horneck, G, Baumstark-Khan, C (eds.), Astrobiology: The Quest for the Conditions of Life. Springer, Berlin, Heidelberg.

4. Altendorf K, Booth IR, Gralla J, Greie J-C, Rosenthal AZ, Wood JM. 2009. Osmotic

Stress. EcoSal Plus 3:10.1128/ecosalplus.5.4.5.

5. Csonka LN. 1989. Physiological and genetic responses of bacteria to osmotic stress. Microbiology and Molecular Biology Reviews 53:121–147.

6. Wood JM, Bremer E, Csonka LN, Kraemer R, Poolman B, van der Heide T, Smith LT. 2001. Osmosensing and osmoregulatory compatible solute accumulation by bacteria. Comparative Biochemistry and Physiology Part A: Molecular & Integrative Physiology 130:437–460.

7. Booth IR, Louis P. 1999. Managing hypoosmotic stress: Aquaporins and medianosensitive channels in *Escherichia coli*. Current Opinion in Microbiology 2:166–169.

8. Inaba M, Sakamoto A, Murata N. 2001. Functional Expression in Escherichia coli of Low-Affinity and High-Affinity Na+(Li+)/H+ Antiporters ofSynechocystis. Journal of Bacteriology 183:1376–1384.

9. Iyer A, Frallicciardi J, le Paige UBA, Narasimhan S, Luo Y, Sieiro PA, Syga L, van den Brekel F, Tran BM, Tjioe R, Schuurman-Wolters G, Stuart MCA, Baldus M, van Ingen H, Poolman B. 2024. The Structure and Function of the Bacterial Osmotically Inducible Protein Y. Journal of Molecular Biology 436:168668.

10. Kim NY, Kim OB. 2024. Oxamic transcarbamylase of Escherichia coli is encoded by the three genes allFGH (formerly fdrA, ylbE, and ylbF). Applied and Environmental Microbiology 90:e00957-24.

11. Kim NY, Kim OB. 2022. The ybcF Gene of Escherichia coli Encodes a Local Orphan Enzyme, Catabolic Carbamate Kinase. J Microbiol Biotechnol 32:1527–1536.

12. 2018. 2017 Verification Monitoring Report, Riverton, Wyoming, Processing Site.

13. McMurdie PJ, Holmes S. 2013. phyloseq: An R Package for Reproducible Interactive Analysis and Graphics of Microbiome Census Data. PLOS ONE 8:e61217.

14. Oksanen J, Blanchet FG, Friendly M, Kindt R, Legendre P, McGlinn D, Minchin PR, O'Hara RB, Simpson GL, Solymos P, Stevens MHH, Szoecs E, Wagner H. 2018. vegan: Community Ecology Package (2.4-6).

15. Palatinszky M, Herbold CW, Sedlacek CJ, Pühringer D, Kitzinger K, Giguere AT, Wasmund K, Nielsen PH, Dueholm MKD, Jehmlich N, Gruseck R, Legin A, Kostan J, Krasnici N, Schreiner C, Palmetzhofer J, Hofmann T, Zumstein M, Djinović-Carugo K, Daims H, Wagner M. 2024. Growth of complete ammonia oxidizers on guanidine. Nature 633:646–653.

SUPPLEMENTAL FIGURES

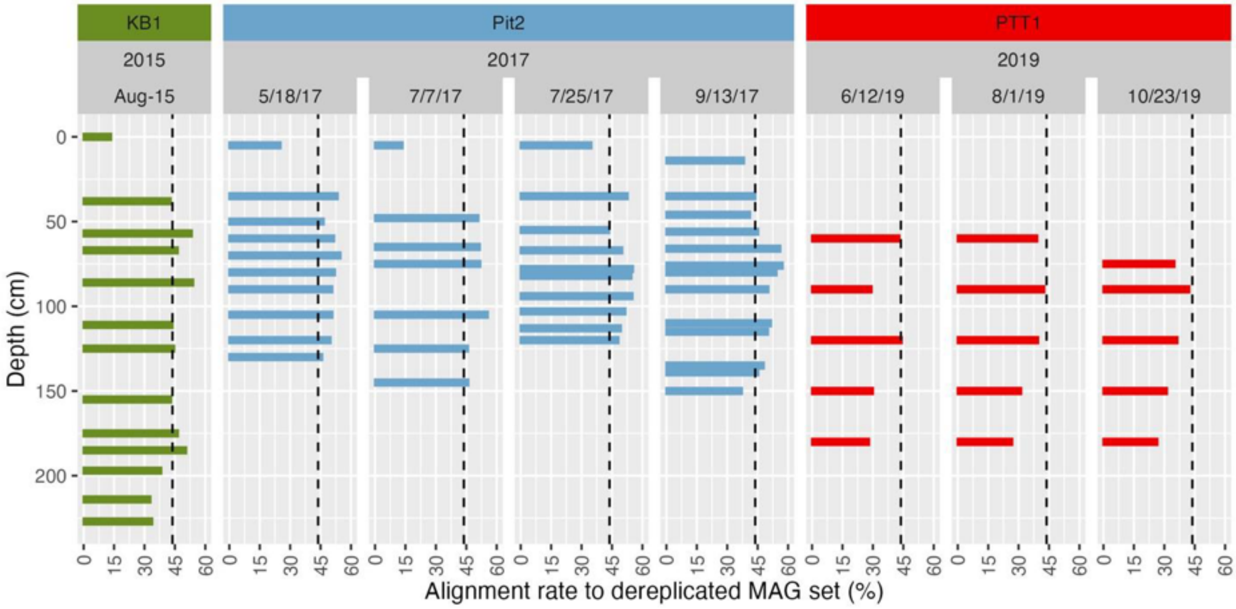

Fig. S1 Percentage of metagenome reads recruited to dereplicated (98% ANI) MAG dataset using bowtie2. Average recruitment rate (43.8%) shown with dashed line.

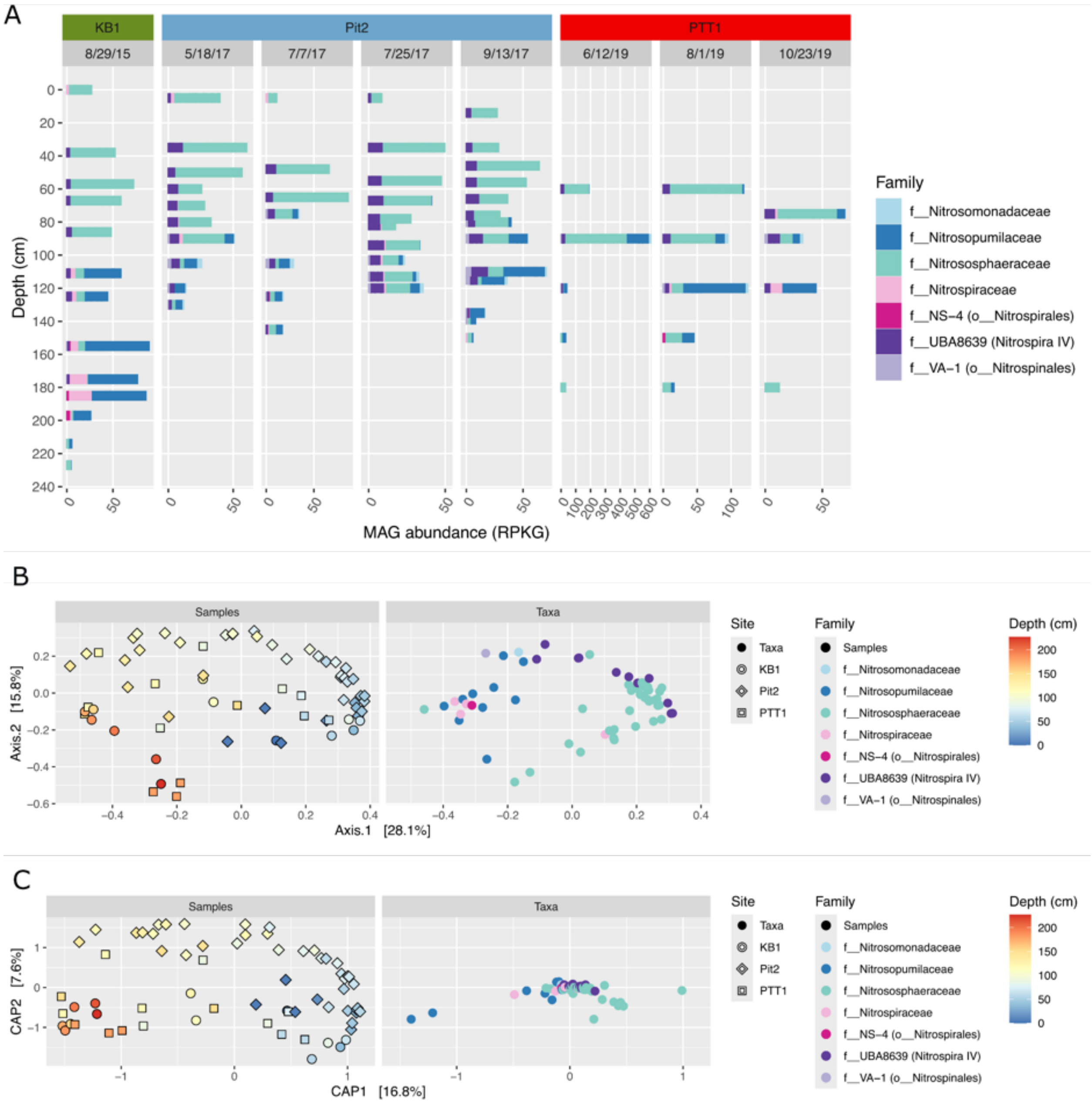

Fig. S2 A. Relative abundance of nitrifier families. B. PCoA biplot of nitrifier community structure based on MAG abundance(distance = bray-curtis). C. CAP biplot of nitrifier community structure based on MAG abundance using depth and site as explanatory variables (distance = bray-curtis).

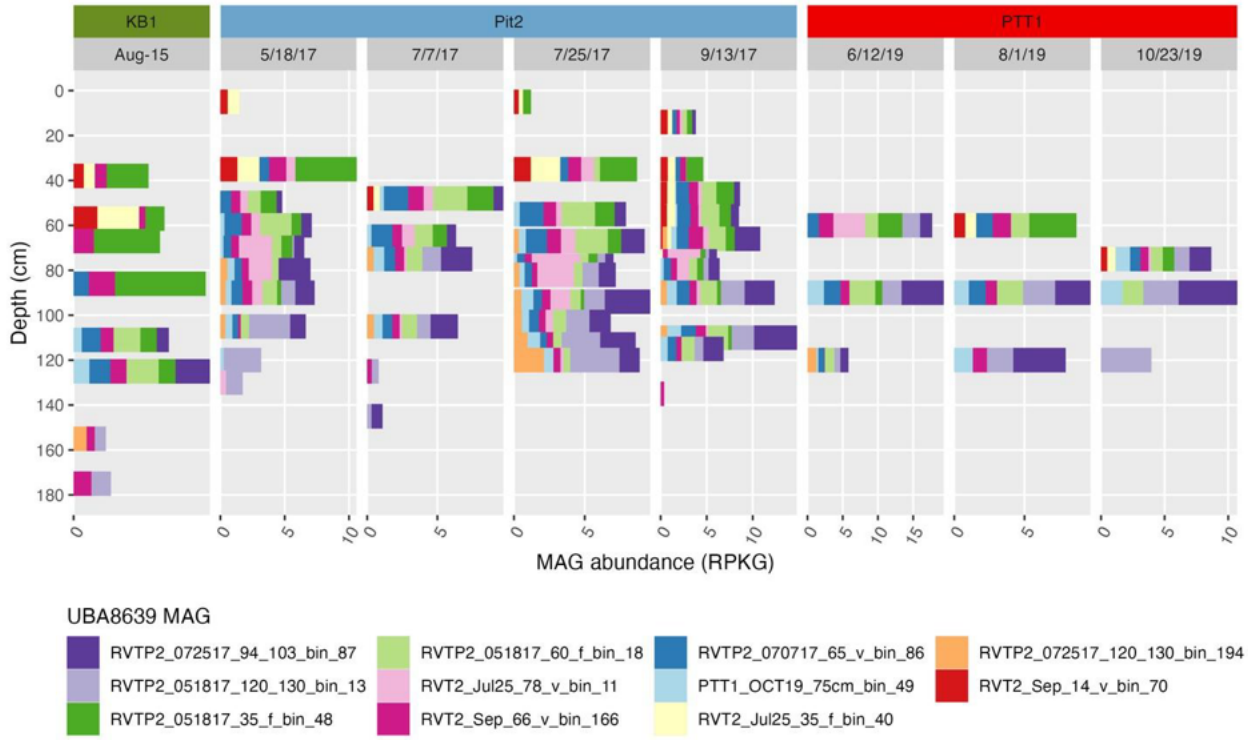

Fig. S3 Relative abundance of lineages (non-redundant MAGs) from the NOB genus UBA8639 (*Nitrospira* lineage IV).

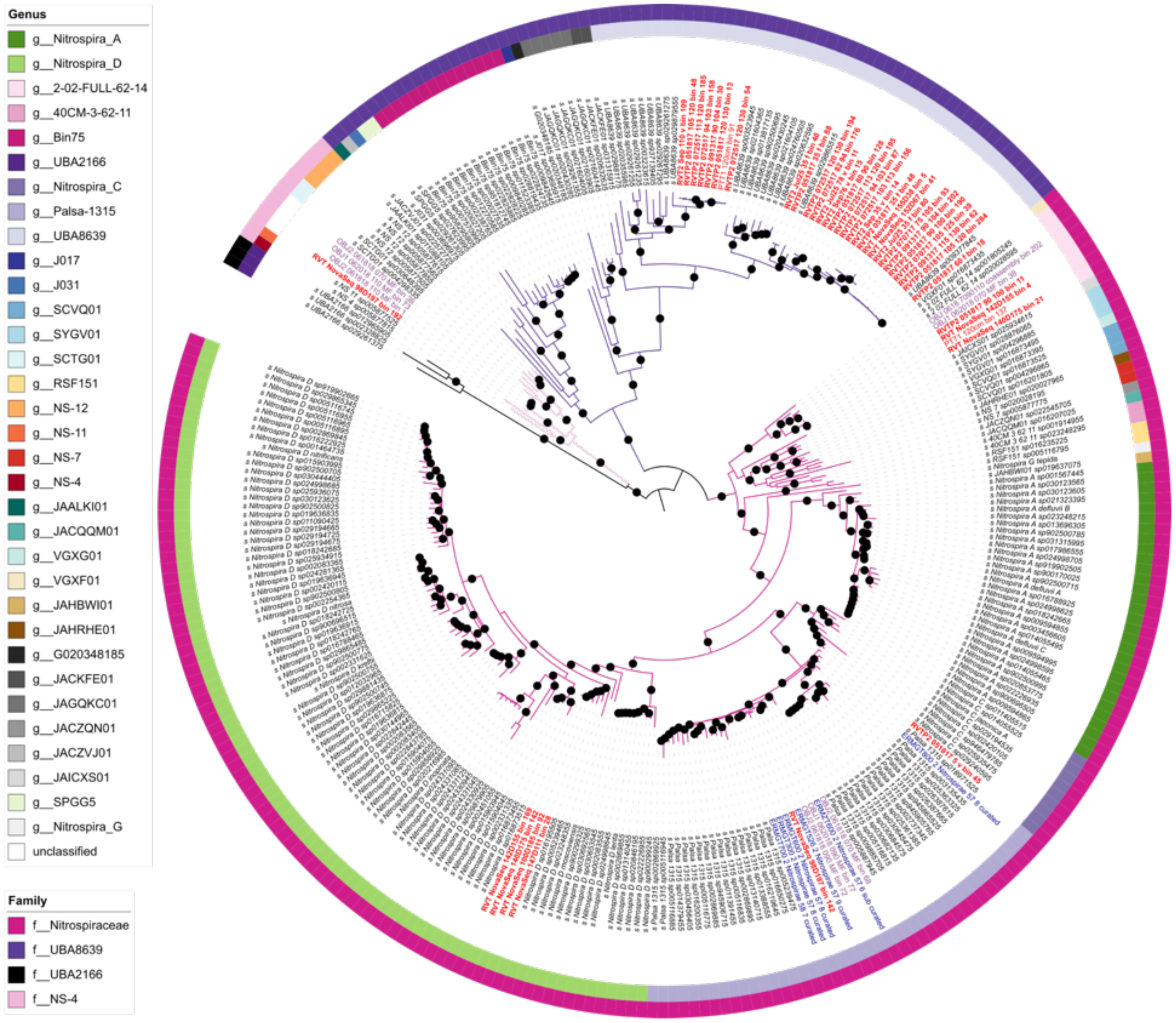

Fig. S4 Concatenated ribosomal tree made using IQ-TREE2 with model JTT+R7 for select *Nitrospirales* MAGs, including *Nitrospirales* MAGs generated from WRB (red), SR (purple), and ER (blue), and NCBI type material (italics). MAGs generated in this study are in bold. Outer ring color indicates GTDB assigned Family and the inner ring indicates Genus. Black dots indicate nodes with >= 90% bootstrap support. Tree is midpoint rooted.

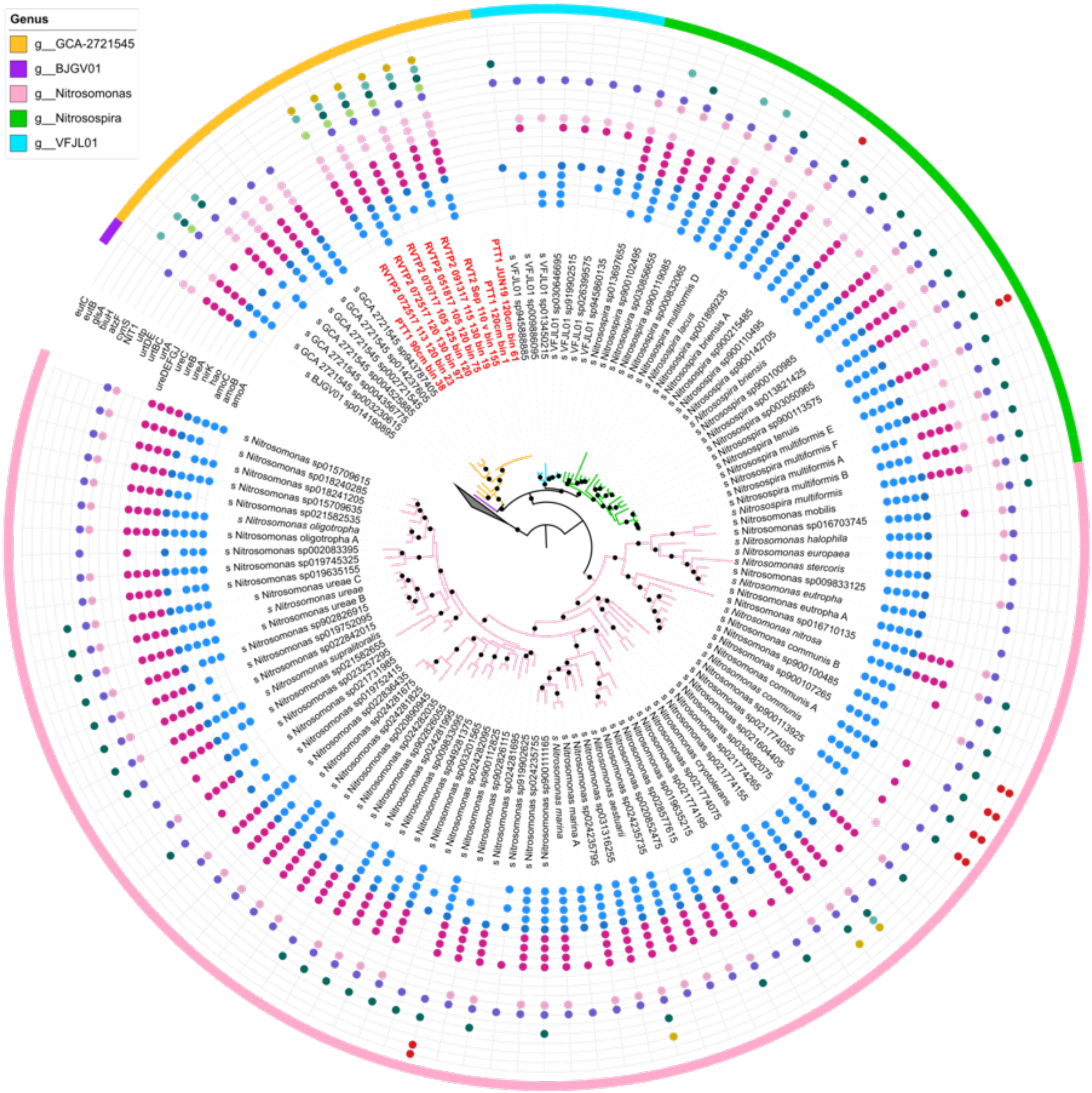

Fig. S5 Concatenated ribosomal tree made using IQ-TREE2 with model JTT+R7 for *Nitrosomonadaceae* MAGs generated from WRB and GTDB species representatives. NCBI type material in italics and MAGs generated in this study are in red and bold. Outer ring color indicates GTDB assigned genus. Black dots indicate nodes with >= 90% bootstrap support. Tree is rooted using *Rhodocyclaceae*. External points indicate gene presence for ammonia monooxygenase (*amoABC*), hydroxylamine oxidoreductase (*hao*), urease (*ure*), urea transport system (*urt*), urea transporter (*utp*), nitrilase (*nit1*), cyanate lyase (*cynS*), allophanate hydrolase (*atzF*), biuret hydrolase (*biuH*), glutaminase (*glsA*), and ethanolamine ammonia lyase (*eutBC*).

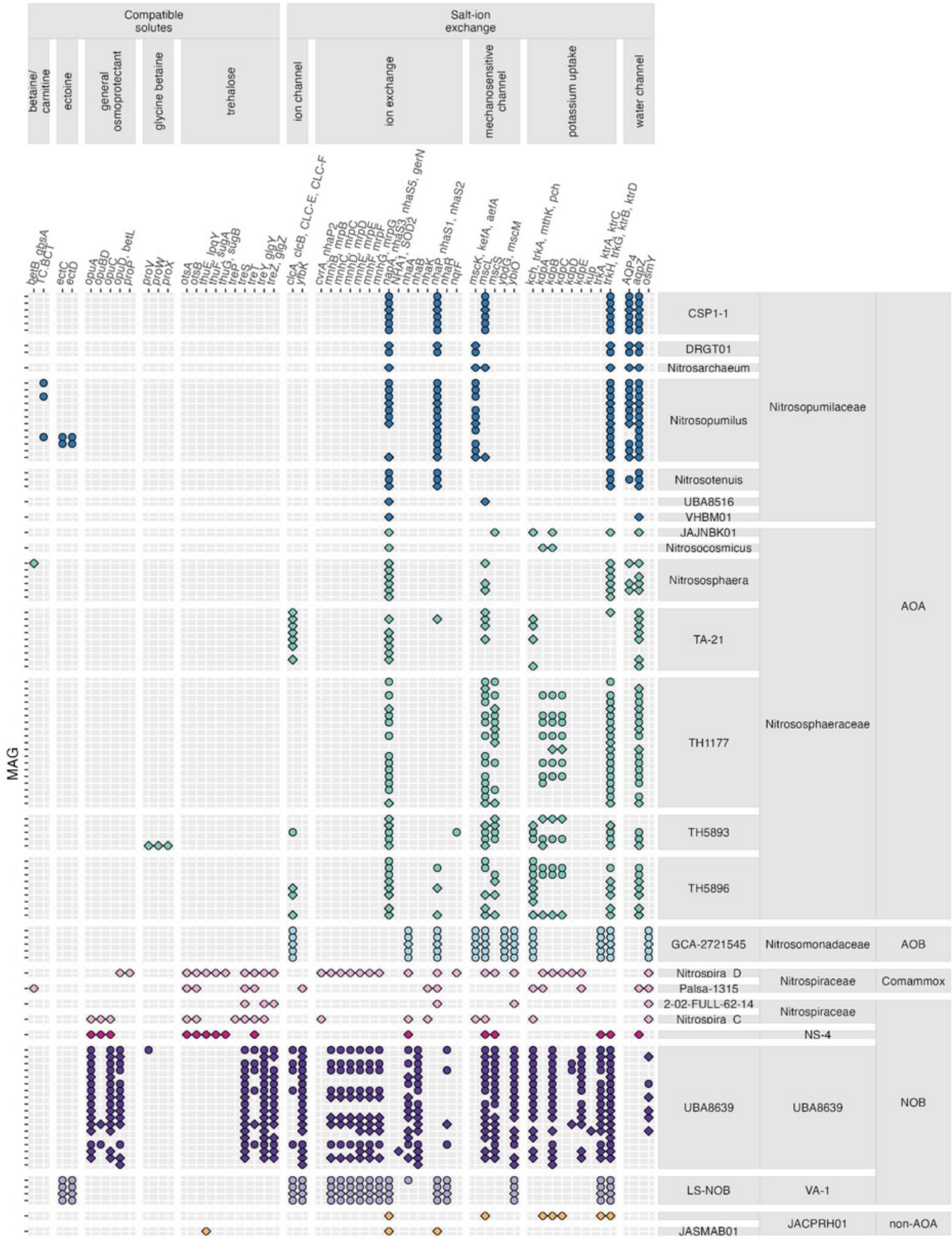

Compatible solutes
Salt-ion exchange
betaine/ carnitine
ectoine
general osmoprotectant
glycine betaine
trehalose
ion channel
ion exchange
mechanosensitive channel
potassium uptake
water channel
betB, gbsA
TC.BCT
ectC
ectD
opuA
opuBD
opuC
opuD
proP, betL
proV
proW
proX
otsA
otsB
thuE, lpqY
thuF, sugA
thuG, sugB
treS
treT
treY, glgY
treZ, glgZ
clcA, clcB, CLC-E, CLC-F
yfbK
cvrA, nhaP2
mnhB, mrpB
mnhC, mrpC
mnhD, mrpD
mnhE, mrpE
mnhF, mrpF
mnhG, mrpG
napA, nhaS5, gerN
NHA1, nhaS3
NHA1, SOD2
nhaA
nhaB
nhaK
nhaP, nhaS1, nhaS2
nhaR
nqrF
mscK, kefA, aefA
mscL
mscS
ybdG, mscM
ybiO
kch, trkA, mthK, pch
kdpA
kdpB
kdpC
kdpD
kdpE
ktrA, ktrC
trkH, trkG, ktrB, ktrD
AQP4
aqpZ
osmY
MAG
CSP1-1
DRGT01
Nitrosarchaeum
Nitrosopumilus
Nitrosotenuis
UBA8516
VHBM01
JAJNBK01
Nitrosocosmicus
Nitrososphaera
TA-21
TH1177
TH5893
TH5896
GCA-2721545
Nitrospira_D
Palsa-1315
2-02-FULL-62-14
Nitrospira_C
UBA8639
LS-NOB
JASMAB01
Nitrosopumilaceae
Nitrososphaeraceae
Nitrosomonadaceae
Nitrospiraceae
Nitrospiraceae
NS-4
UBA8639
VA-1
JACPRH01
AOA
AOB
Comammox
NOB
non-AOA

Fig. S6 Osmoregulation gene presence and absence in representative MAGs (diamonds) and MAGs > 90% complete. Genes include: betaine-aldehyde dehydrogenase (*betB*), betaine/carnitine transporter (TC.BCT), ectoine synthase (*ectCD*), osmoprotectant transport system (*opuABCD*), glycine betaine transporter (*opuD*), proline/betaine transporter (*proP*), glycine betaine/proline transport system (*proVWX*), trehalose 6-phosphate synthase (*otsAB*), trehalose transport system (*thuEFG*), chloride channel (*clcA, yfbK*), potassium/hydrogen antiporter (cvrA/nhaP2), sodium/hydrogen antiporter [Mhn-type (*mhnB*-*G*), Nha-type (*nhaAB*), low affinity (*nhaP/nhaS1*), and high affinity (*napA*/*nhaP3*)], transcriptional activator of *nhaA* (*nhaR*), monovalent cation/hydrogen antiporter (*nhaK*), sodium-transporting NADH:ubiquinone oxidoreductase (*nqrF*), mechanosensitive channel [potassium-dependent mechanosensitive channel (*mscK*/*kefA*), miniconductance (*mscM*), small conductance (*mscS*), large conductance (*mscL*), moderate conductance (*ybiO*)], voltage gated potassium channel (*kch*), potassium-transporting ATPase (*kdpABC*), KDP operon response regulator (*kdpDE*), Tkr/Ktr potassium channel (*tkrAB*/*ktrAB*), KUP system potassium uptake (*kup*), aquaporin 4 (AQP4), aquaporin Z (*aqpZ*), and osmotically induced protein (*osmY*).

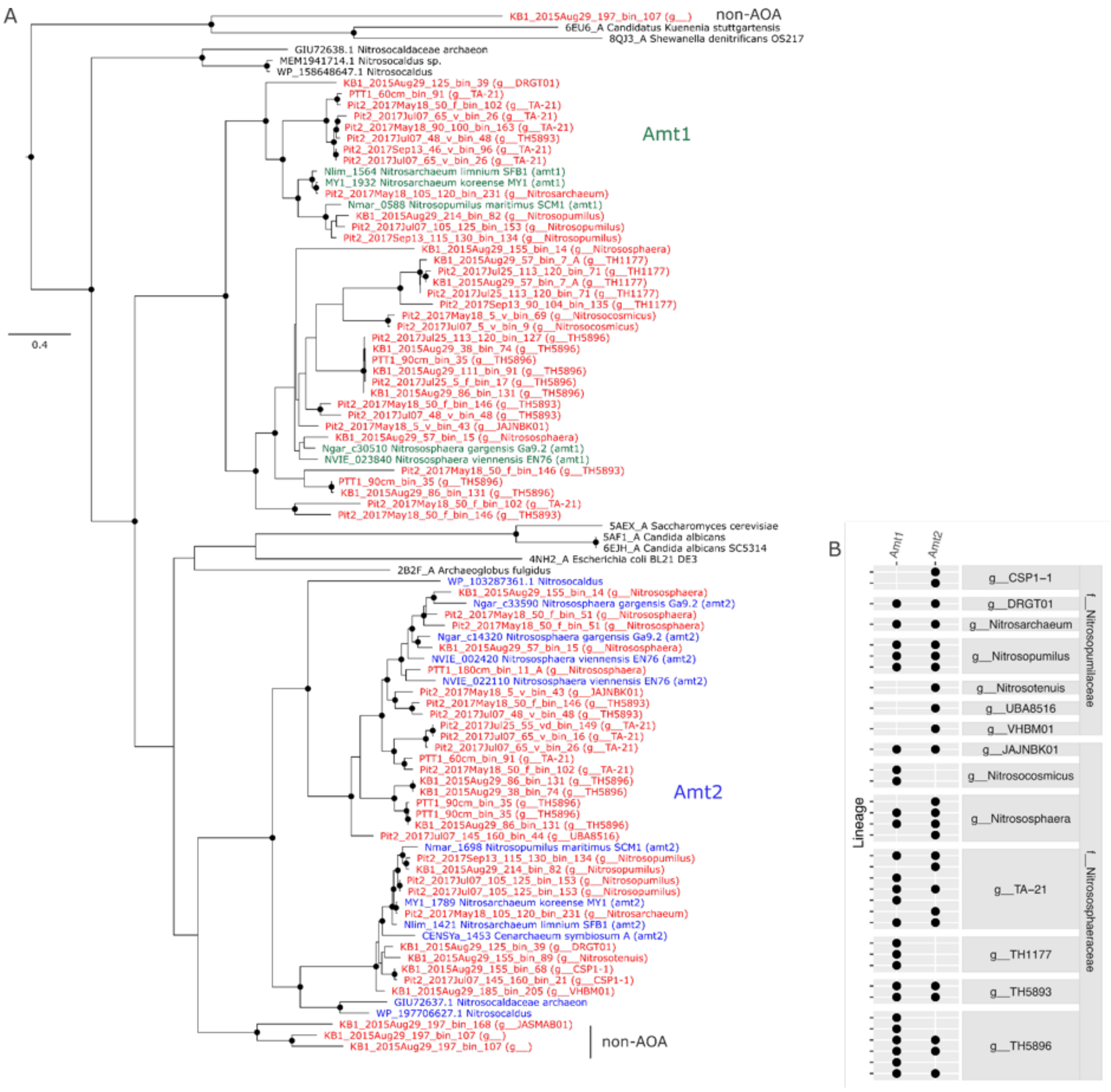

Fig. S7 A. Phylogeny of *Nitrososphaeria* ammonium transporter (*amt*) genes based on an amino acid alignment made with IQ-TREE2 model LG+F+R5. Protein sequences from MAGs generated in this study are in red. Black dots indicate bootstrap of >= 90%. Tree is midpoint rooted. B. The presence of low-affinity (Amt1) and high-affinity (Amt2) ammonium transporter genes in AOA lineages.

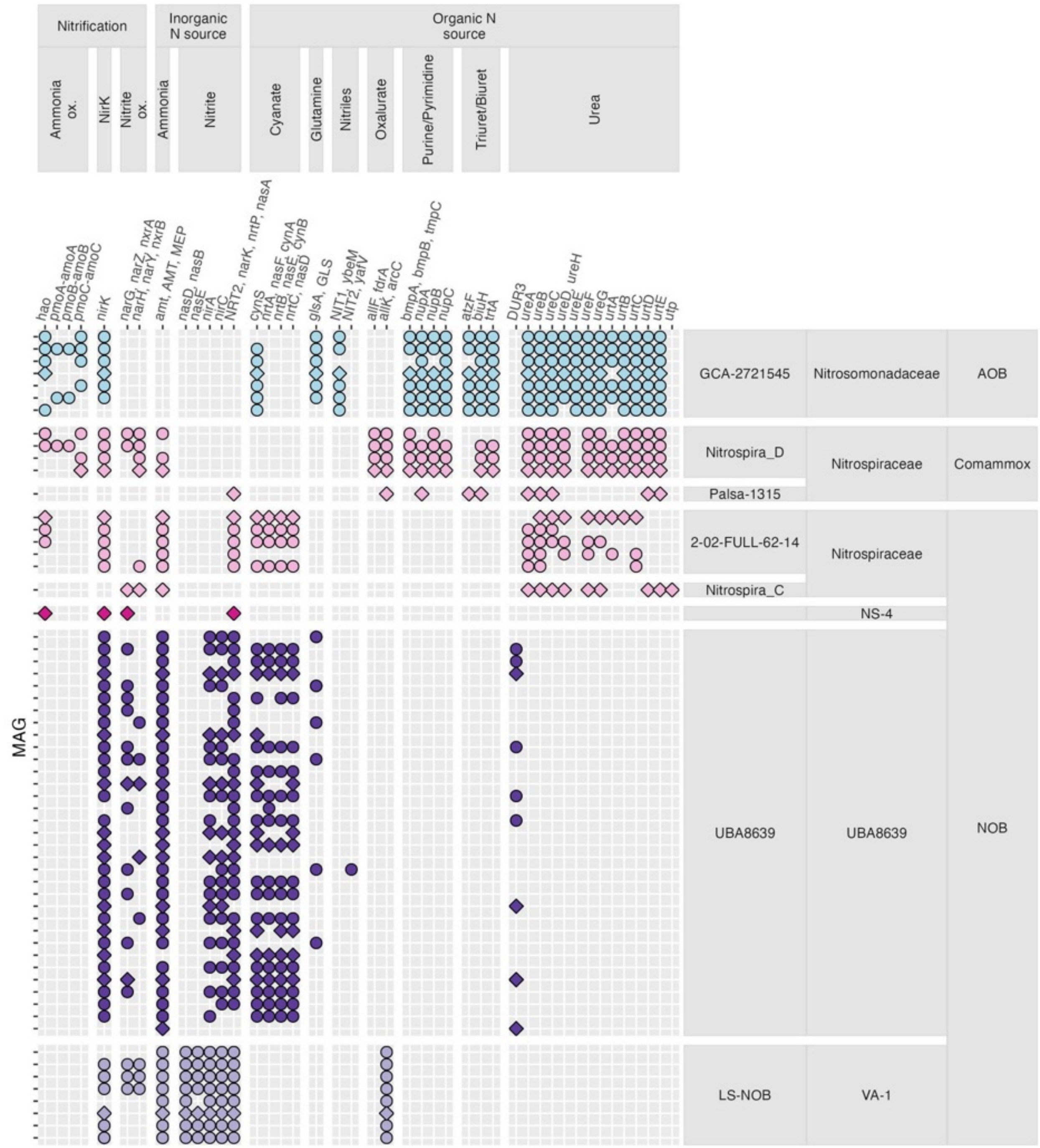

Fig. S8 Gene presence/absence for bacterial nitrifier lineage representatives (diamonds) and MAGs > 70% complete (circles). Genes include: ammonia monooxygenase (*amoABC*), hydroxylamine oxidoreductase (*hao*), nitrite oxidoreductase (*nxrAB*), nitrite reductase (*nirK*), ammonium transporter (*amt*), nitrite transporter (*nirC*), NNP family nitrate/nitrite transporter (*narK*), nitrite reductase (*nirA, nasBDE*), allophanate hydrolase (*atzF*), biuret hydrolase (*biuH*), triuret hydrolase (*trtA*), uncharacterized *gatA*-like amidohydrolase, ABC-type nucleoside transporter (*bmpA*, *nupABC*), cyanate lyase (*cynS*), nitrite/nitrate/cyanate transporter

(*nrtABCD/cynABD*), glutaminase (*glsA*), nitrilase (*nit1*), nitrile hydratase (*nthA*), omega amidase (*nit2*), oxamic transcarbamylase (*allFGH*), carbamate kinase (*allK*), urease (*ure*), urea transport system (*urt*), urea transporter (*utp*), urea-proton symporter (*DUR3*).

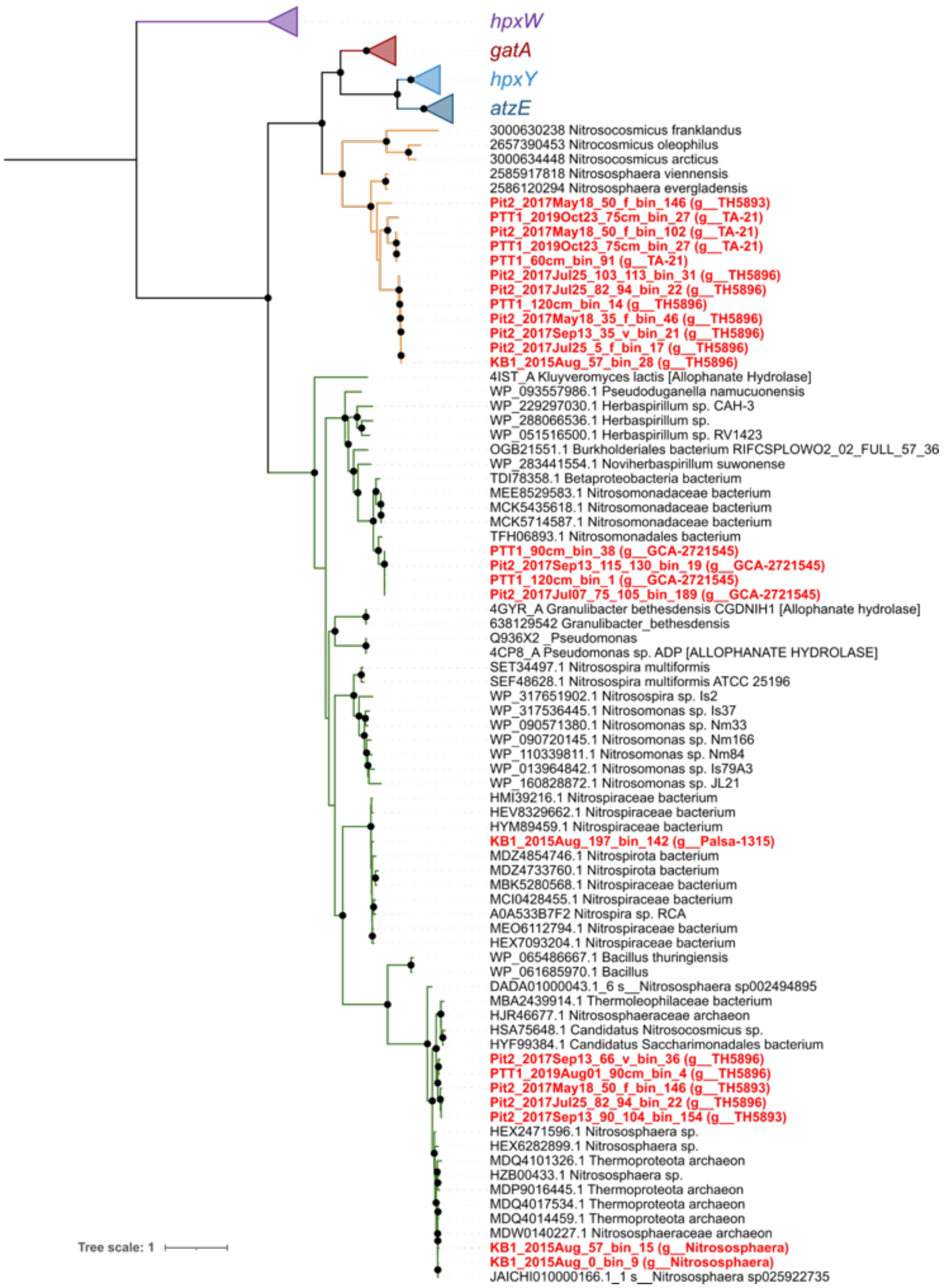

Fig. S9 Phylogeny of amidohydrolase genes based on a 533 position amino acid alignment of 150 sequences made with IQ-TREE2 model LG+G4. Gene sequences from MAGs generated in this study are in bold red. Black dots indicate bootstrap of >= 90%. Tree is midpoint rooted. Structurally characterized proteins in brackets. Genes include allophanate hydrolase (*atzF*, green branches), carboxybiuret hydrolase (*atzE*), glutamyl-tRNA amidotransferase (*gatA*),

oxamate carbamoyltransferase (*hpx*Y), oxamate amidohydrolase (*hpxW),* and the uncharacterized *“gatA-like”* amidohydrolase from AOA (orange branches).

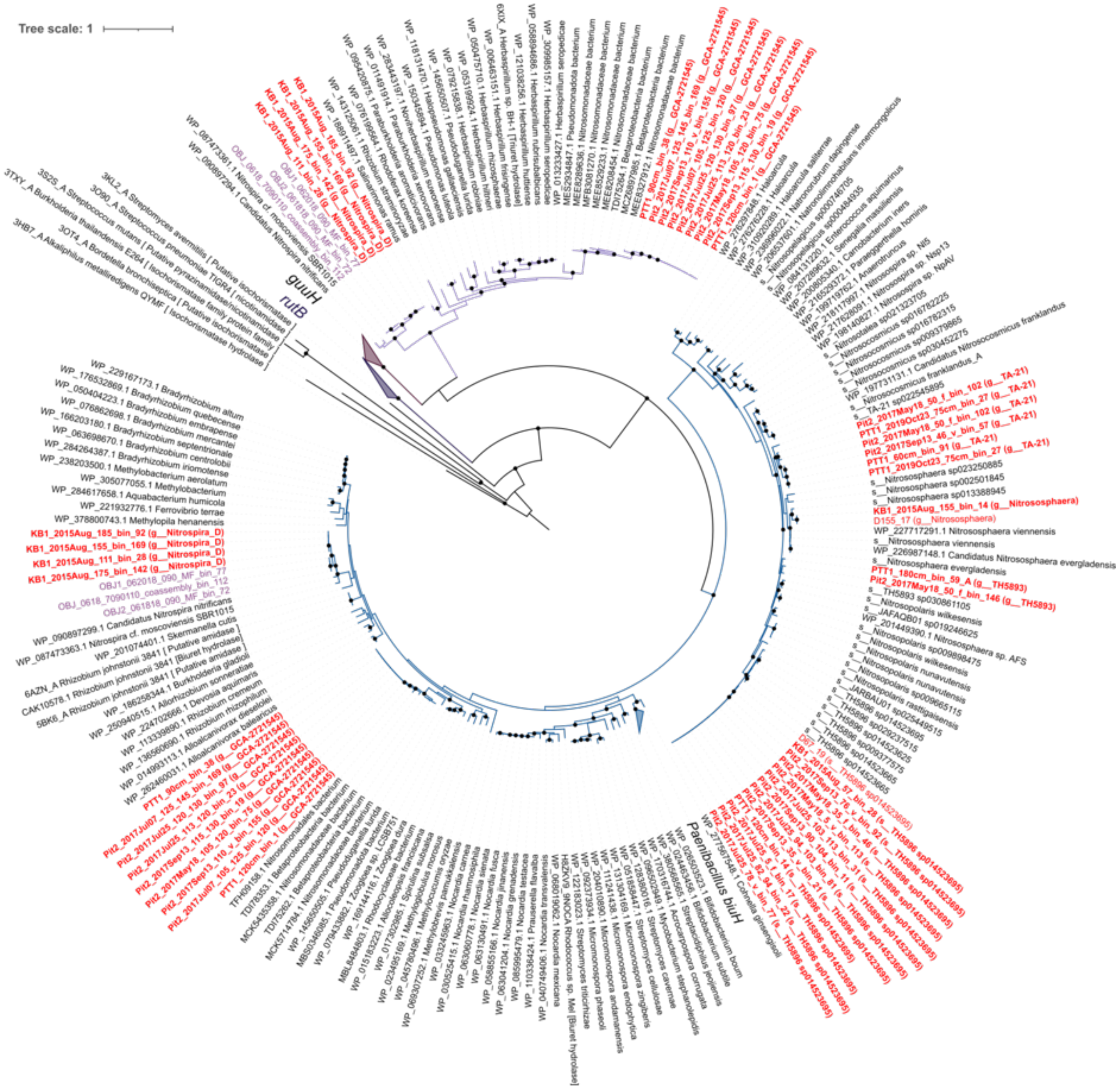

Fig. S10 Phylogeny of isochorismatase genes based on a 257 position amino acid alignment of 234 sequences made with IQ-TREE2 model LG+R6. Gene sequences from MAGs generated in this study are in bold red. Black dots indicate bootstrap of >= 90%. Tree is midpoint rooted and pruned to exclude distantly related structurally characterized isochorismatase-family proteins. Genes shown include biuret hydrolase (*biuH*), triuret hydrolase (*trtA*), guanylurea hydrolase (*guuH*),and ureidoacrylate amidohydrolase (*rutB*).

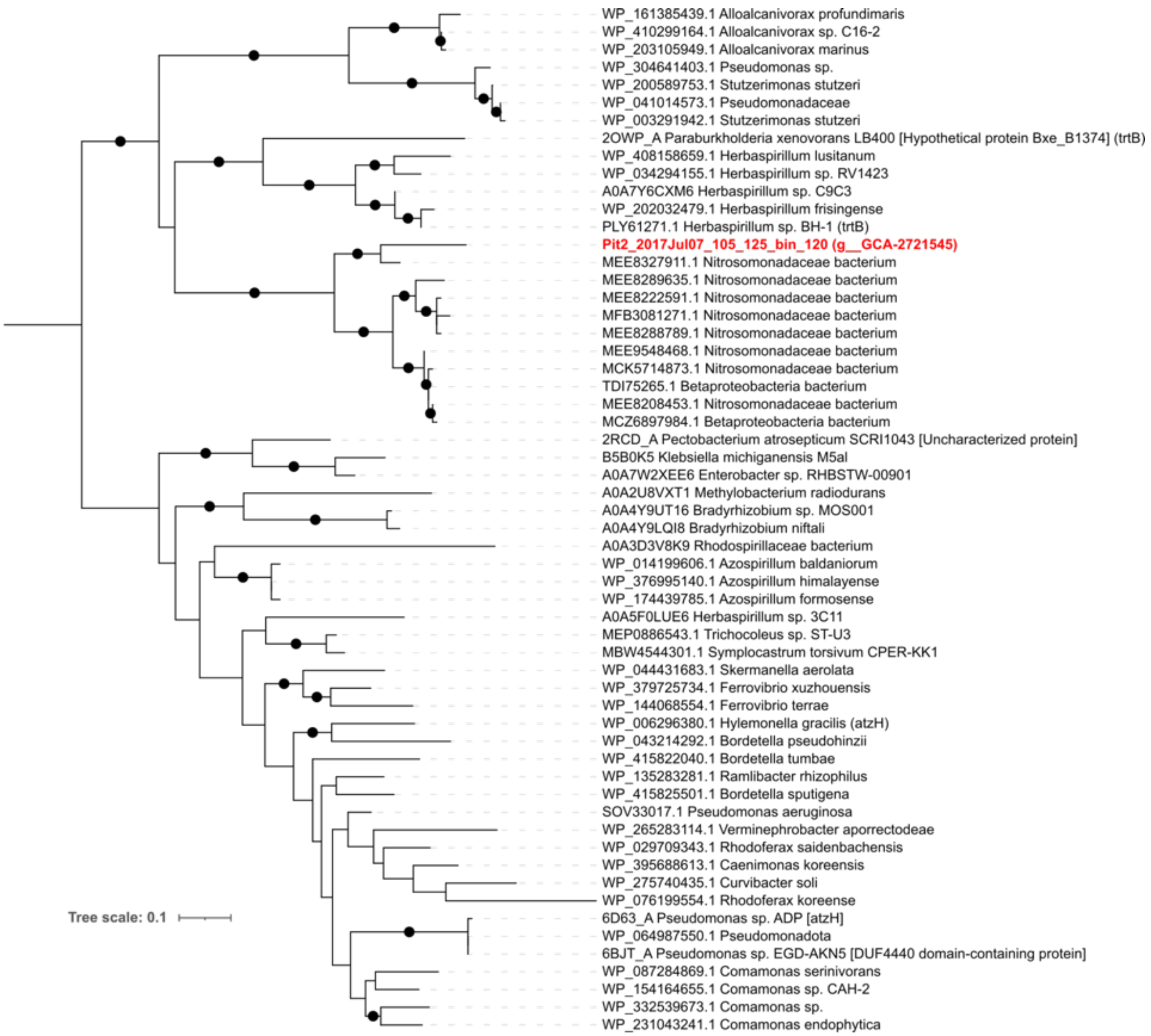

Fig. S11 Phylogeny of decarboxylase genes based on a 126 position amino acid alignment of 13 sequences made with IQ-TREE2 model WAG+I+G4. Gene sequences from MAGs generated in this study are in bold red. Black dots indicate bootstrap of >= 90%. Tree is midpoint rooted. Genes include carboxybiuret decarboxylase (*trtB*), dicarboxyurea decarboxylating amidohydrolase (*atzH*), and decarboxylating amidohydrolase (*hpxZ).*

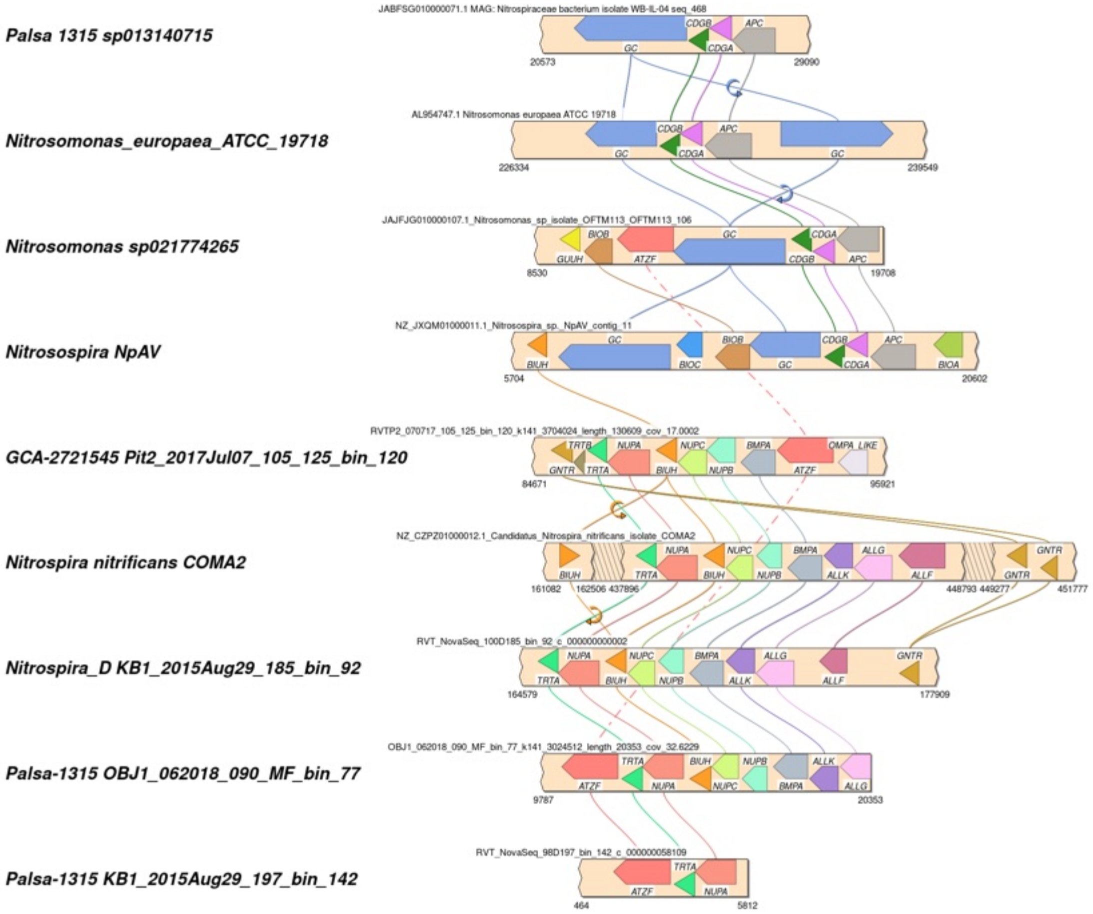

Fig. S12 Gene synteny for WRB bacterial ammonia oxidizer contigs encoding *biuH* and reference genomes encoding guanidine decarboxylase [see (15)] and/or biuret hydrolase. Annotated genes include: guanidine carboxylase (GC), carboxyguanidine deiminase (*cdgAB*), amino acid/polyamine/organocation permease superfamily permease (APC), guanylurea hydrolase (*guuH*), allophanate hydrolase (*atzF*), biotin synthase (*bioB*), malonyl-CoA O-methyltransferase (*bioC*), adenosylmethionine-8-amino-7-oxononanoate aminotransferase (*bioA*), biuret hydrolase (*biuH*), triuret hydrolase (*trtA*), carboxybiuret decarboxylase (*trtB*), ABC-type nucleoside transporter (*bmpA-nupABC*), oxamic transcarbamylase (*allFGH)*, catabolic carbamate kinase (*allK*), *gntR* family transcriptional regulator, major outer membrane protein (*ompA*).

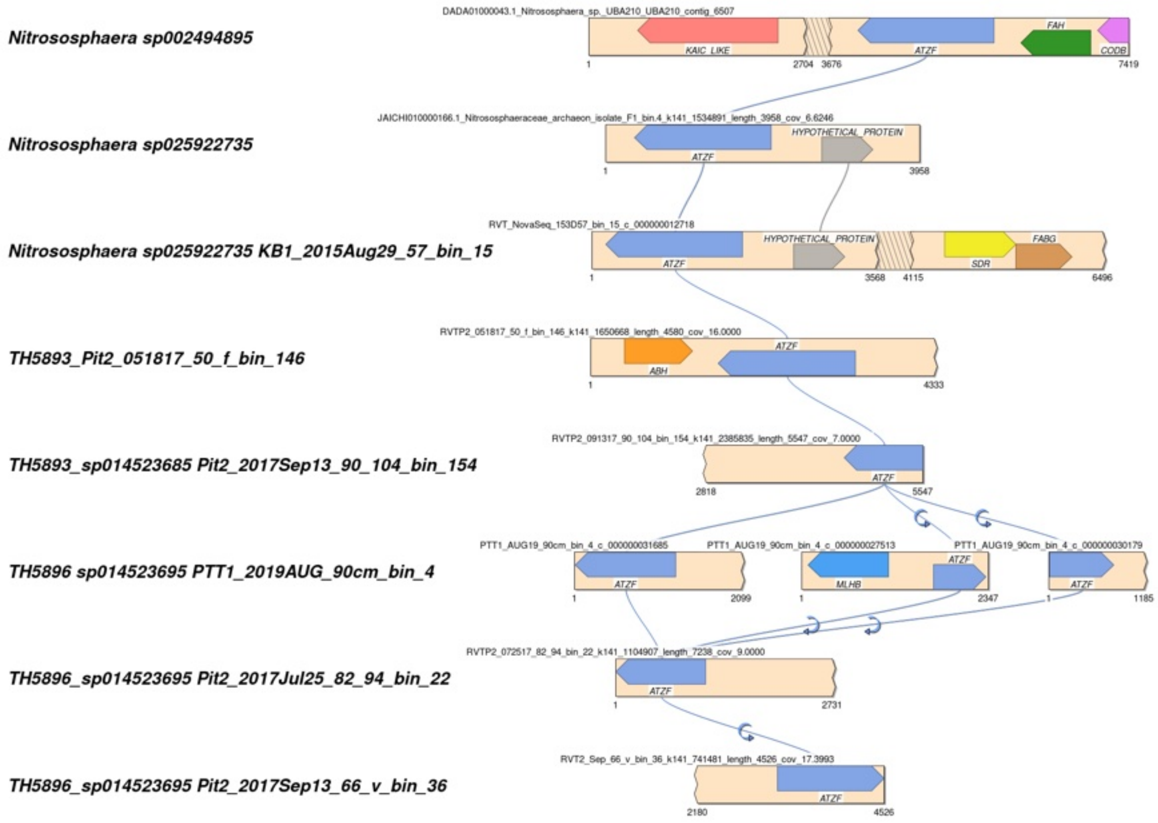

Fig. S13 Gene synteny for AOA contigs encoding allophanate hydrolase (*atzF*). Genes include cytosine permease (*codB*), fumarylacetoacetate hydrolase family protein (FAH), circadian clock protein (*kaiC*), epsilon-lactone hydrolase (*mlhB*), alpha beta hydrolase (AB), SDR family oxidoreductase, and 3-oxoacyl-acyl-carrier protein reductase (*fabG*). Alignment cutoff used was 25% due to small contig size.

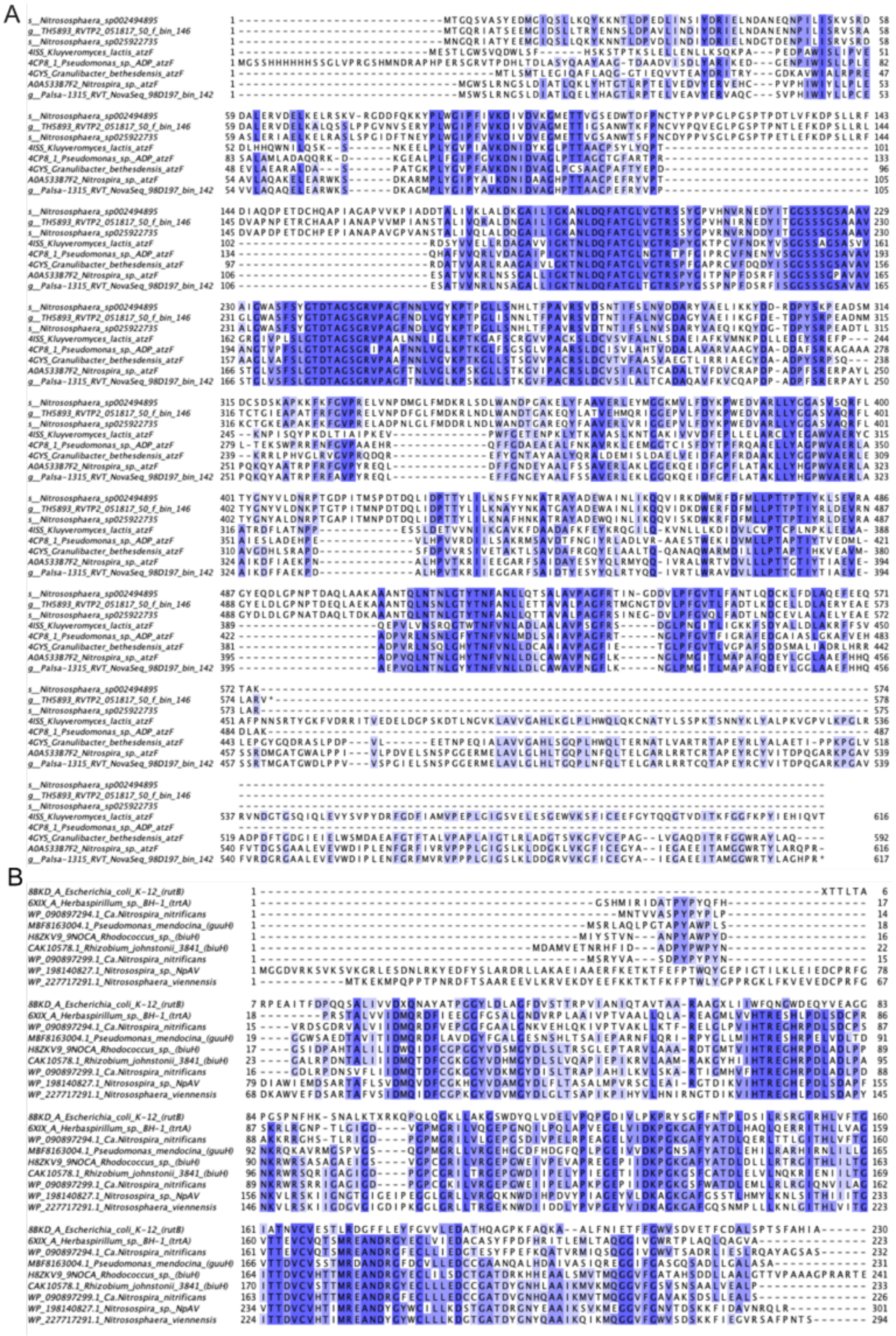

Fig. S14 A) Amidohydrolase (e.g., *atzF*, *atzE*, *gatA*, etc) and B) isochorismatase-like (e.g., *biuH*, *trtA*, *guuH*, etc) hydrolase amino acid alignments for select sequences from WRB MAGs and structurally characterized proteins.

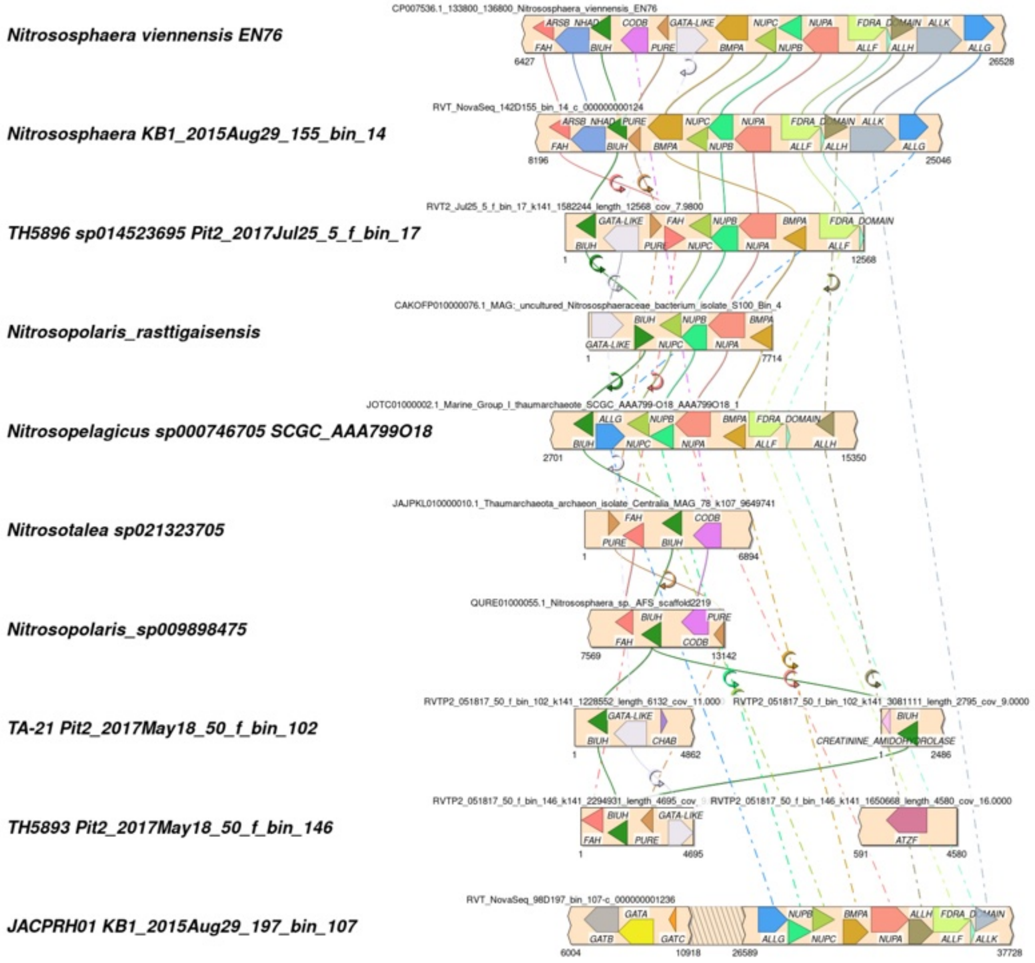

Fig. S15 Gene synteny for *Nitrososphaeria* contigs encoding *biuH* including a non AOA lineage (JACPRH01). Annotated genes include allophanate hydrolase (*atzF*), biuret hydrolase (*biuH*), ABC-type nucleoside transporter (*bmpA-nupABC*), oxamic transcarbamylase (*allFGH)*, catabolic carbamate kinase (*allK*), cytosine permease (*codB*), intermolecular transferase 5-(carboxyamino)imidazole ribonucleotide mutase (*purE*), fumarylacetoacetate hydrolase family protein (FAH), creatininase, uncharacterized amidohydrolase (*gatA*-like), cation transport regulator (*chaB*), glutamyl-tRNA amidotransferase (*gatCAB*).

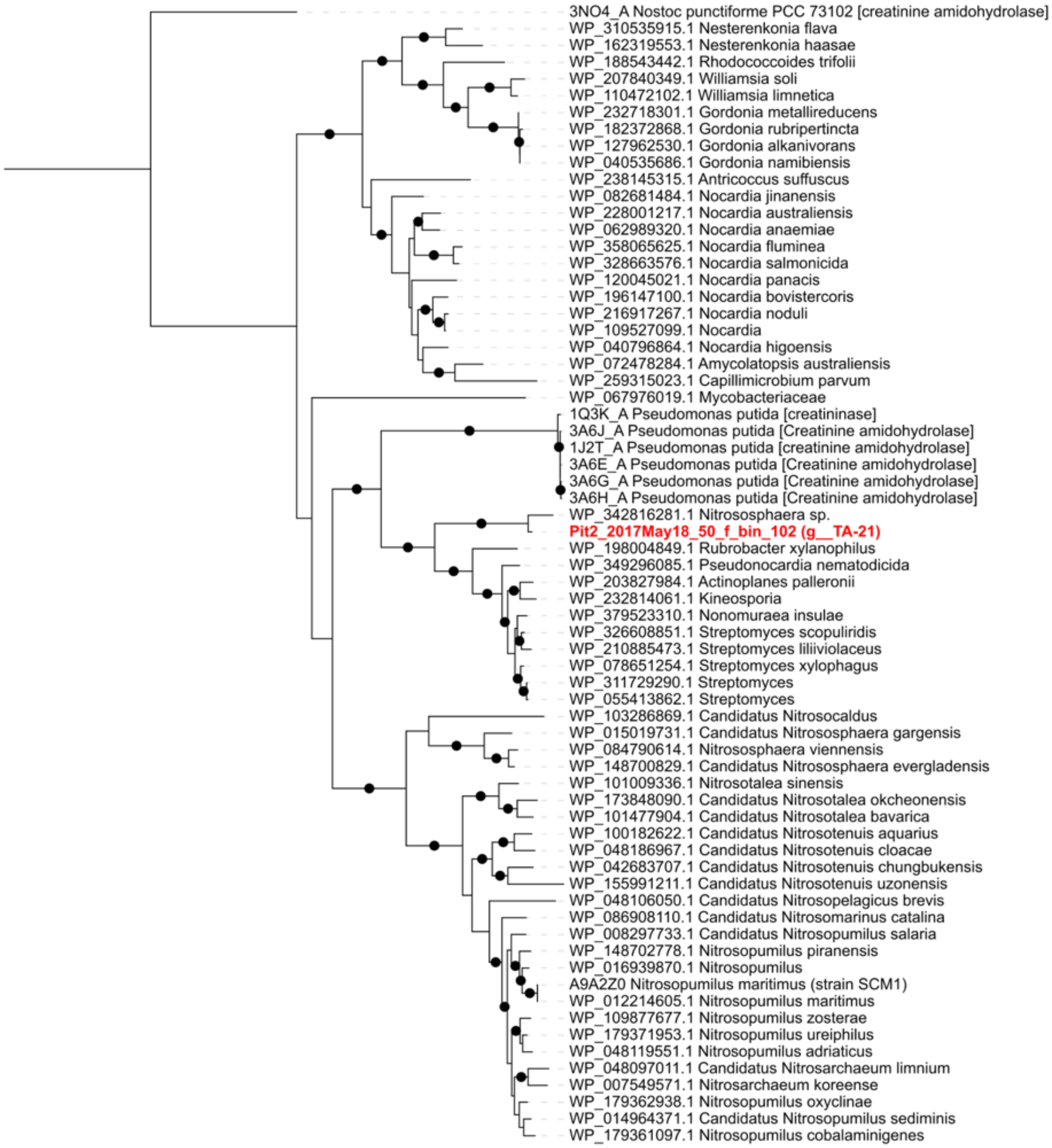

Fig. S16 Phylogeny of creatininase based on a 307 position amino acid alignment of 68 sequences made with IQ-TREE2 model WAG+R4. Gene sequences from MAGs generated in this study are in bold red. Black dots indicate bootstrap of >= 90%. Tree is midpoint rooted. Structurally characterized proteins in brackets.

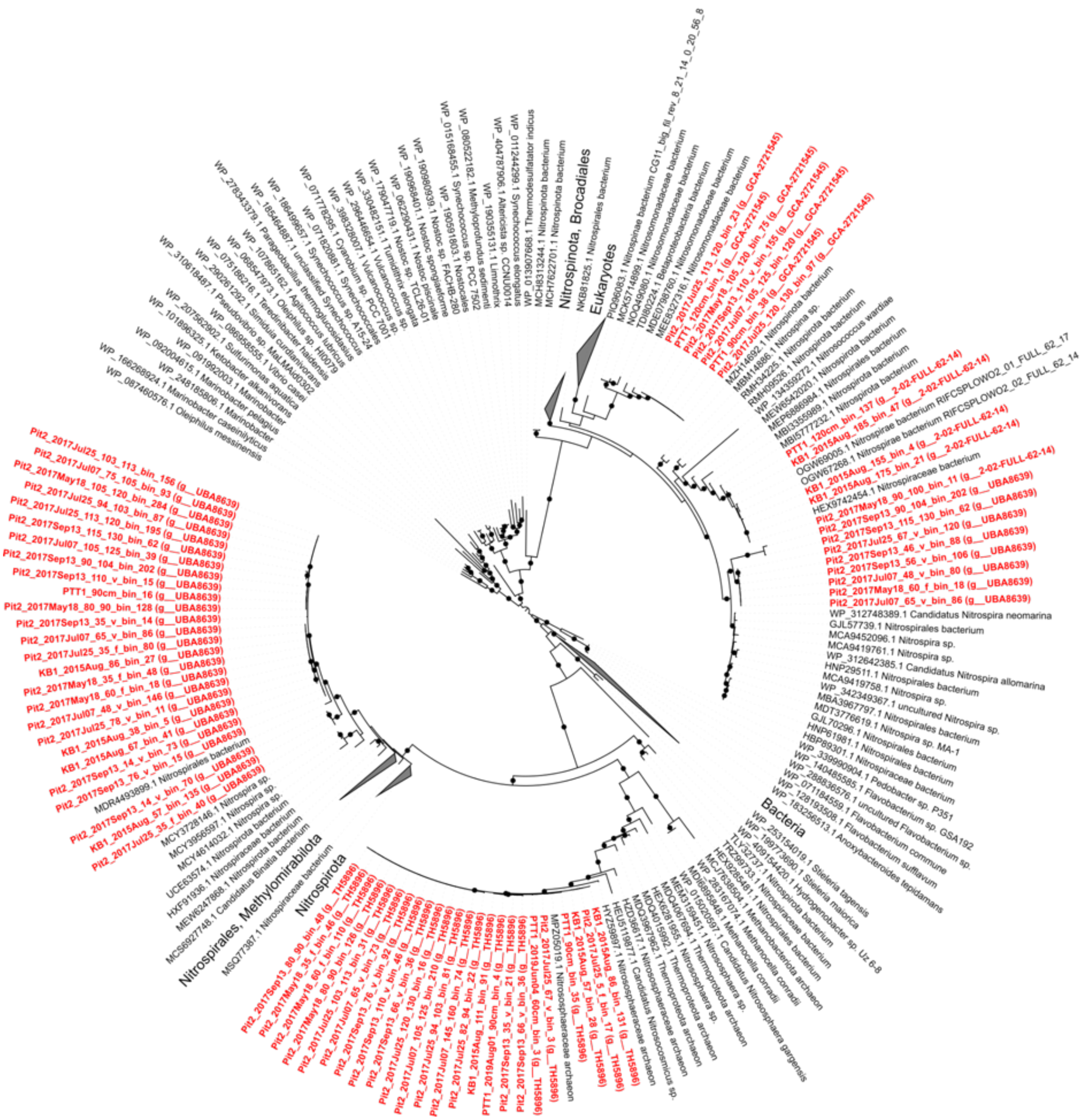

Fig. S17 Phylogeny of cyanate lyase (*cynS*) based on a 157 position amino acid alignment of 392 sequences made with IQ-TREE2 model LG+R6. Gene sequences from MAGs generated in this study are in bold red. Black dots indicate bootstrap of >= 90%. Tree is midpoint rooted.

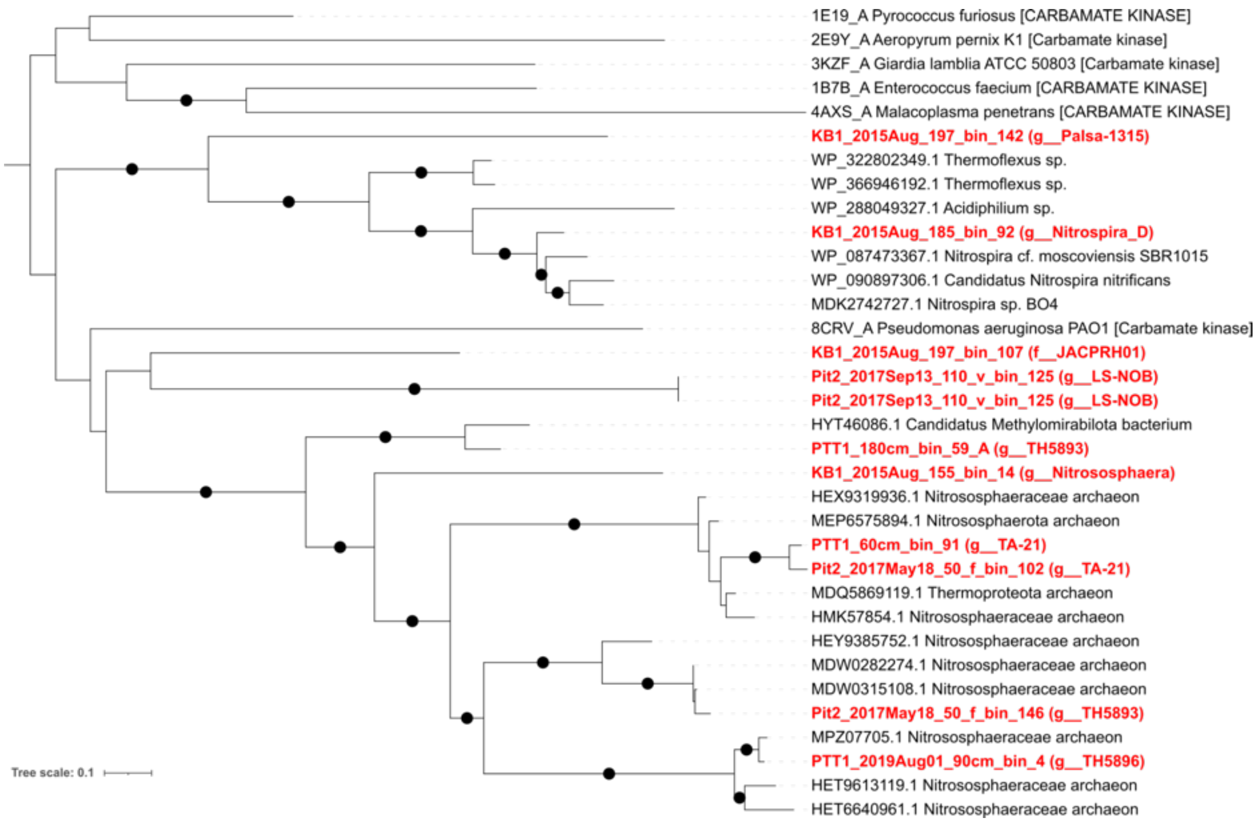

Fig. S18 Phylogeny of carbamate kinase (*allK*) based on a 647 position amino acid alignment of 34 sequences made with IQ-TREE2 model LG+I+G4. Gene sequences from dereplicated MAGs generated in this study are in bold red. Black dots indicate bootstrap of >= 90%. Tree is midpoint rooted. Structurally characterized proteins in brackets.

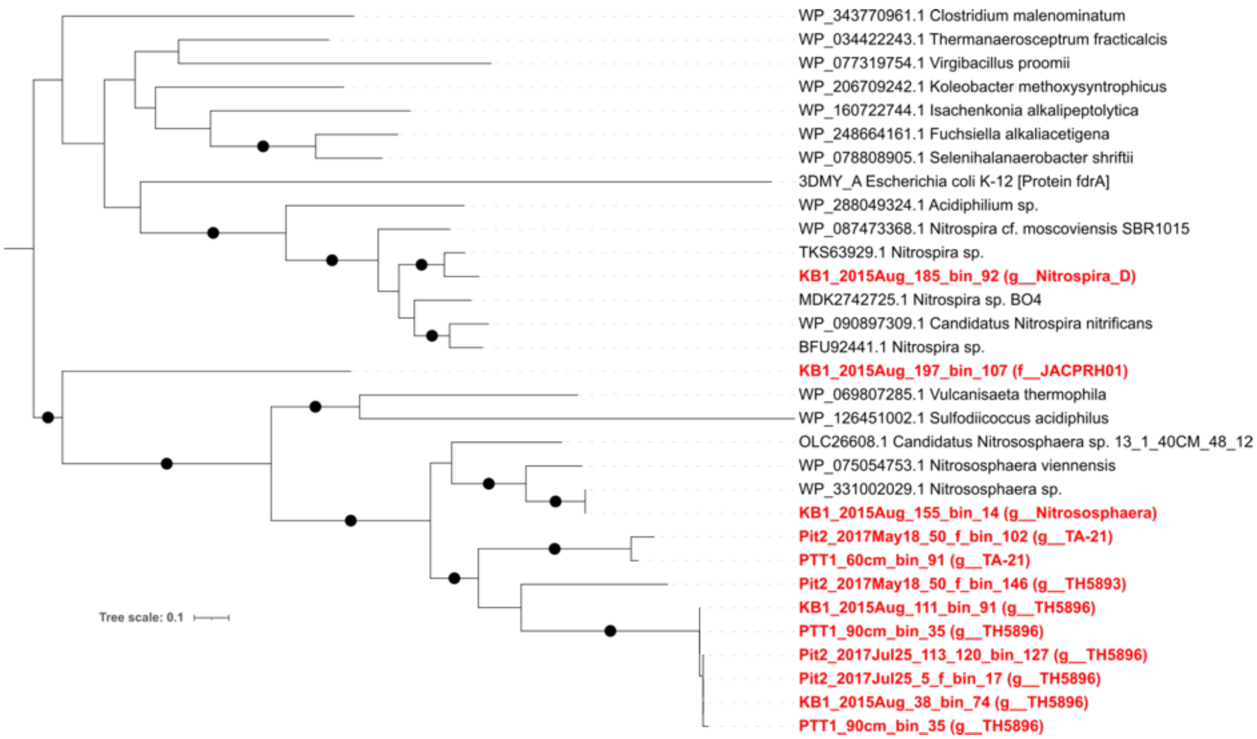

Fig. S19 Phylogeny of oxamate transcarboxylase (*allF*) based on a 465 position amino acid alignment of 31 sequences made with IQ-TREE2 model LG+I+G4. Gene sequences from dereplicated MAGs generated in this study are in bold red. Black dots indicate bootstrap of >= 90%. Tree is midpoint rooted.

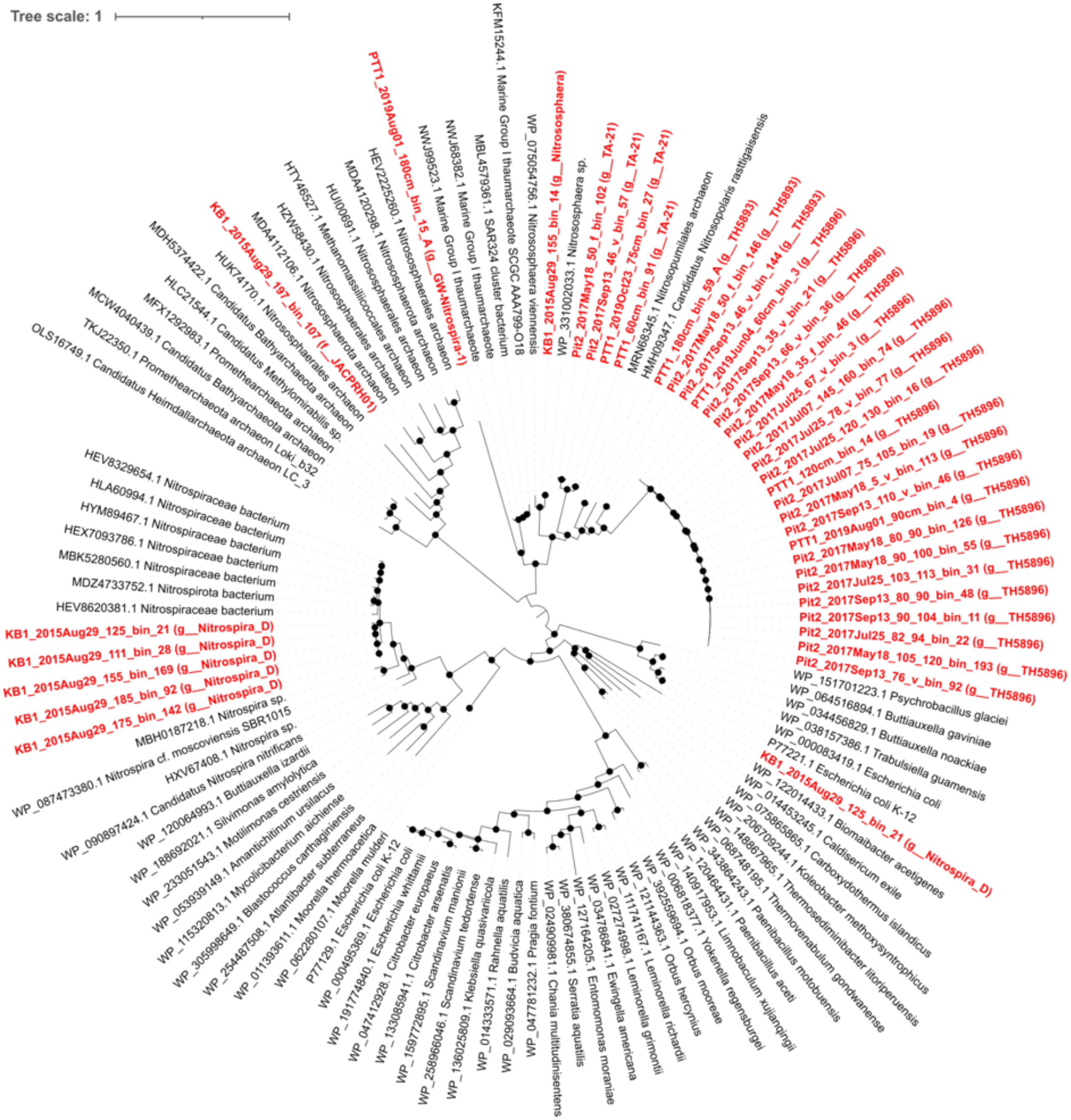

Fig. S20 Phylogeny of oxamate transcarboxylase (*allG*) based on a 428 position amino acid alignment of 113 sequences made with IQ-TREE2 model LG+R5. Gene sequences from MAGs generated in this study are in bold red. Black dots indicate bootstrap of >= 90%. Tree is midpoint rooted.

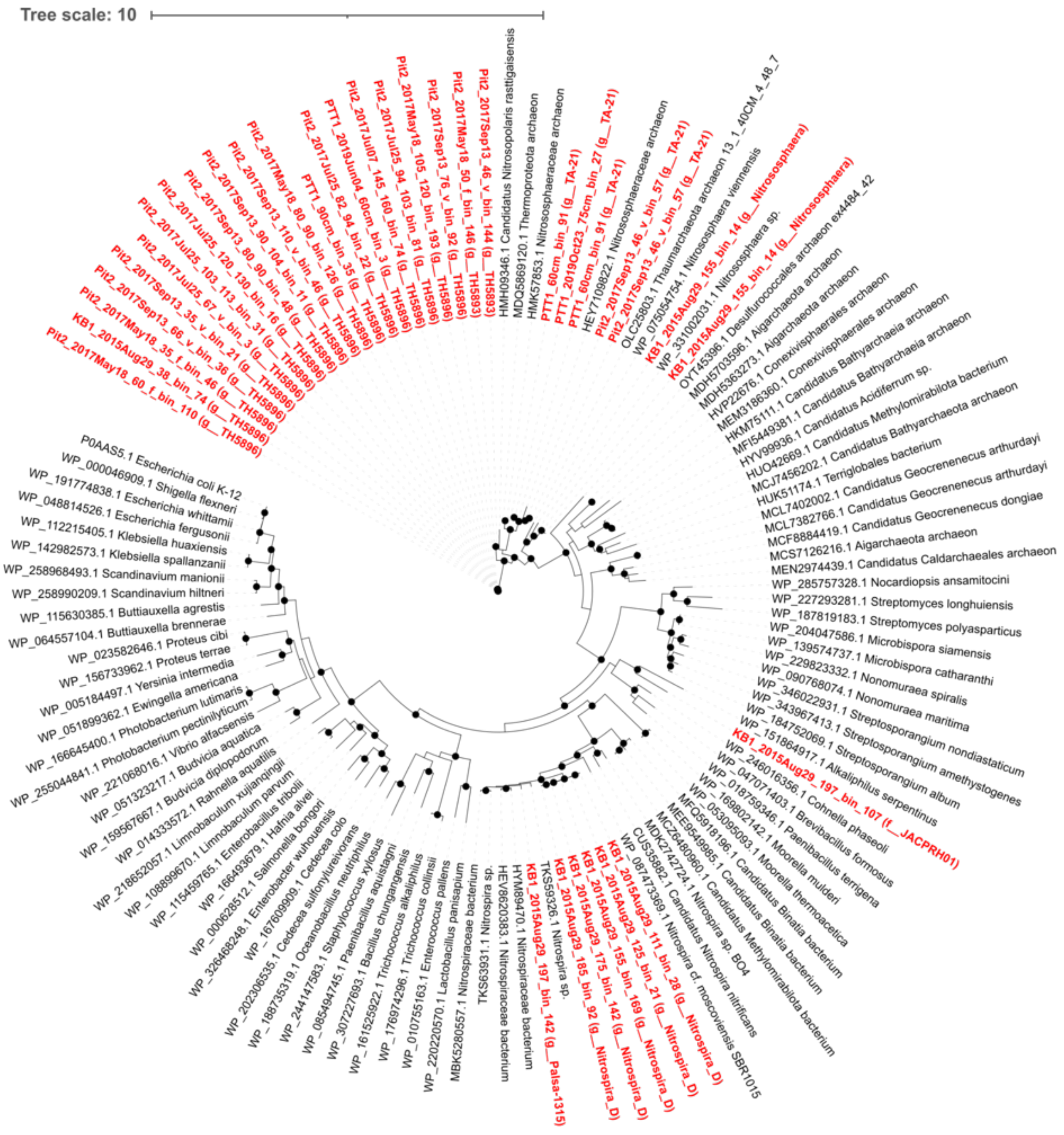

Fig. S21 Phylogeny of oxamate transcarboxylase (*allH*) based on a 452 position amino acid alignment of 121 sequences made with IQ-TREE2 model WAG+F+R6. Gene sequences from MAGs generated in this study are in bold red. Black dots indicate bootstrap of >= 90%. Tree is midpoint rooted.
